# The impact of ambient air pollution on hospital admissions, length of stay and hospital costs for patients with diabetes mellitus and comorbid respiratory diseases in Panzhihua, Southwest China

**DOI:** 10.7189/jogh.13.04118

**Published:** 2023-10-13

**Authors:** Xianzhi Li, Bin Yu, Yajie Li, Haorong Meng, Meiying Shen, Yan Yang, Zonglei Zhou, Shunjin Liu, Yunyun Tian, Xiangyi Xing, Li Yin

**Affiliations:** 1 Meteorological Medical Research Center, Panzhihua Central Hospital, Panzhihua, Sichuan Province, China; 2 Clinical Medical Research Center, Panzhihua Central Hospital, Panzhihua, Sichuan Province, China; 3 Dali University, Dali, Yunnan Province, China; 4 Institute for Disaster Management and Reconstruction, Sichuan University - Hong Kong Polytechnic University, Chengdu, Sichuan Province, China; 5 Tibet Center for Disease Control and Prevention, Lhasa, Tibet Autonomous Region, China; 6 Yunnan Center for Disease Control and Prevention, Kunming, Yunnan Province, China; 7 Nursing department, Panzhihua Central Hospital, Panzhihua, Sichuan Province, China; 8 Department of Respiratory and Critical Care Medicine, Panzhihua Central Hospital, Panzhihua, Sichuan Province, China; 9 Department of Epidemiology, School of Public Health, Fudan University, Shanghai, China; 10 Department of Pharmacy, Panzhihua Central Hospital, Panzhihua, Sichuan Province, China

## Abstract

**Background:**

There is limited evidence on association between air pollutants and hospital admissions, hospital cost and length of stay (LOS) among patients with diabetes mellitus (DM) and comorbid respiratory diseases (RD), especially in low- and middle-income countries (LMICs) with low levels of air pollution.

**Methods:**

Daily data on RD-DM patients were collected in Panzhihua from 2016 to 2020. A generalised additive model (GAM) was used to explore the effect of air pollutants on daily hospital admissions, LOS and hospital cost. Attributable risk was employed to estimate RD-DM's burden due to exceeding air pollution exposure, using both 0 microgrammes per cubic metre (μg/m^3^) and WHO’s 2021 air quality guidelines as reference.

**Results:**

For each 10 ug/m^3^ increase of particles with an aerodynamic diameter <2.5 micron (μm) (PM_2.5_), particles with an aerodynamic diameter <10 μm (PM_10_), sulfur dioxide (SO_2_), nitrogen dioxide (NO_2_) and ozone (O_3_), the admissions of RD-DM patients increased by 7.25% (95% CI = 4.26 to 10.33), 5.59% (95% CI = 3.79 to 7.42), 10.10% (95% CI = 7.29 to 12.98), 12.33% (95% CI = 8.82 to 15.95) and -2.99% (95% CI = -4.08 to -1.90); per 1 milligramme per cubic metre (mg/m^3^) increase of carbon monoxide (CO) corresponded to a 25.77% (95% CI = 17.88 to 34.19) increment for admissions of RD-DM patients. For LOS and hospital cost, the six air pollutants showed similar effect. Given 0 μg/m^3^ as the reference, NO_2_ showed the maximum attributable fraction of 32.68% (95% CI = 25.12 to 39.42%), corresponding to an avoidable burden of 5661 (95% CI = 3611 to 5860) patients with RD-DM.

**Conclusions:**

There is an association between PM_2.5_, PM_10_, SO_2_, NO_2_, and CO with increased hospital admissions, LOS and hospital cost in patients with RD-DM. Disease burden of RD-DM may be improved by formulating policies related to air pollutants exposure reduction, especially in LMICs with low levels of air pollution.

Respiratory diseases (RD) and diabetes mellitus (DM) are major health concerns, which have contributed to a huge burden on society [1]. The Global Burden of Disease (GBD) Study 2019 suggested that chronic obstructive pulmonary disease (COPD), a serious chronic RD, was responsible for 2638.2 global age-standardised point prevalence and 42.5 deaths per 100 000 population [2], and the global age-standardised point prevalence and death rates for type 2 diabetes were 5282.9 and 18.5 per 100 000 [3]. Numerous studies have recently confirmed the pathophysiology of the metabolic glycaemic disorder and the RD with similar mechanism shared by the two conditions [4,5]. As a major comorbidity of several RD [6], DM affect the disease behaviour of patients with RD. Conversely, patients with DM are at high risk of asthma, COPD, etc. [7]. Moreover, the same pathophysiological mechanisms determining the major degenerative complications of DM may further lead to pulmonary function deficiency [5]. Given the heavy burden caused by RD and comorbid DM [8], it is crucial to explore risk factors that help to develop appropriate measures to reduce the prevalence of RD and DM comorbidity.

The GBD 2019 showed that the largest increases in risk exposure from 2010 to 2019 were ambient particulate matter pollution, drug use, high fasting plasma glucose, and high body-mass index [9]. Ambient air pollutants are one of modifiable risk factors for both RD and DM [10]. Epidemiological investigations have provided a large amount of evidence indicating the association between short-term and lifelong particulate matter (PM) and ozone (O_3_) exposure and RD [9,11-13]. Previous studies also have linked air pollutants exposure with DM [14,15]. However, few studies have examined the impact of air pollution on RD and comorbid DM [16]. Furthermore, almost all of them focus on cities with large economies, and severe air pollution, such as Shanghai [17], Bangkok [18] and Beijing [19]. Studies have shown that differences in the toxic composition of air pollutants and climate conditions across regions may influence the incidence rate of RD and DM [20,21]. Thus, evidence from low-pollution areas is needed. Air pollution has recently attracted attention for its effect on hospital admissions, hospital length of stay (LOS), and hospital costs [22]. Compared to traditional indices such as mortality and morbidity, the three indices can intuitively reflect the disease burden.

Despite air quality has improved over the past few years, China still has a high RD burden caused by ambient air pollution [23], as well as increasing prevalence of DM [16]. Located in southwest China, Panzhihua is considered an ideal place to explore the health effects of air pollution in low-pollution areas due to its good air quality [24]. This study aimed to conduct a time-series analysis to estimate the association of air pollutants (including particles with an aerodynamic diameter <2.5 micron (μm) (PM_2.5_), particles with an aerodynamic diameter <2.5 μm (PM_10_), sulfur dioxide (SO_2_), nitrogen dioxide (NO_2_), carbon monoxide (CO), and ozone (O_3_)) with hospital admissions, LOS and hospital cost for RD-DM patients in Panzhihua. Besides, the attributable risk of RD-DM was estimated due to excess air pollution exposure using different reference concentration.

## METHODS

### Study area

Panzhihua, located in southwest China, covers about 7414 km^2^ and has 1.12 million permanent residents. With its location in the upper Yangtze River valley, the city has a typical dry-hot valleys (DHVs) climate characterised by high temperature and low humidity. In recent years, Panzhihua's air quality has been much better than most metropolises. In spite of this, the major ambient particulate pollutants in Panzhihua like PM_2.5_ still exceed the concentration limits recommended by 2021 WHO's Air Quality Guidelines (AQG) [25]. The study area included all districts and counties in Panzhihua.

### Daily records of hospital admissions for RD-DM

Daily records of hospital admissions between 1 January 2016 and 31 December 2020 were collected from Panzhihua Health Information Center (http://wjw.panzhihua.gov.cn/jgsz/zsdw/). Each hospitalisation record was first extracted for the following information: ID, admission date, residential address, age, sex, LOS, hospital cost and primary diagnosis; then using hospitalisation records reshaped into time series data, we obtained RD-DM patients' daily admissions, LOS and hospital costs. All diagnoses were coded using the International Classification of Diseases, 10th Revision (ICD-10). Patients with an ICD-10 diagnosis of both J00-J99 and E10-E14 were defined as having RD-DM.

### Air pollution and meteorology data

We have acquired daily mean concentrations of PM_2.5_, PM_10_, SO_2_, NO_2_, CO, O_3_ from 1 January 2016 to 31 December 2020 from the Panzhihua Environmental Monitoring Center (http://sthjj.panzhihua.gov.cn/). And we obtained daily weather data from the Panzhihua Meteorological Bureau (http://sc.cma.gov.cn/ds/pzh/), including relative humidity and average temperature. We matched air pollution and meteorology data with date-specific hospital admissions.

### Consumer price index (CPI)

In this study, we collected information on Panzhihua's CPI for the period of 2016-2020 from the Panzhihua Bureau of Statistics (http://tjj.panzhihua.gov.cn/).

### Statistical methods

#### Descriptive statistics

Mean, standard deviation (SD) and quartiles were used to describe the distribution of patient data, air pollutants, and meteorology factors. Besides, to show the daily variation in hospital admissions, LOS and hospital cost for RD-DM patients, the calendar heat map was employed. The correlation between air pollution and meteorological factors was estimated using Spearman rank correlation analysis.

#### Estimating associations

An over-dispersed generalised additive models (GAM) with a quasi-Poisson link was applied to estimate the associations between air pollutants and hospital admissions for RD-DM due to daily hospital admissions usually following a Poisson distribution [26]. We found daily LOS and hospital cost were approximately normally distributed based on our data; thus, a GAM with a Gaussian link was utilised to detect the short impact of air pollution on LOS and hospital cost.

Several potential confounding factors were controlled by cubic spline functions in the GAM model, including day of week (DOW), relative humidity, average temperature, public holidays and long-term trends and seasonality. According to previous research, the degree of freedom (*df*) was determined to be seven per year for long-term trends and seasonality [16,27] and three for mean temperature and relative humidity [28,29]. We included DOW and holidays as categorical variables in model. CPI is an index number measuring the average price of consumer goods and services purchased by households, with the percent change in the CPI commonly used as a measure of inflation [30,31]. It is suggested that the CPI is related to the hospitalisation expenditure of the population [31] and we thus considered the CPI for hospital cost.

Considering the delayed effect and acute effect of air pollution, single-day lag (from current day to seven days before: lag0-lag7) and multi-day moving average (from lag01 to lag07) was used to identify lag patterns in ambient air pollution [16]. According to previous literatures [32-35], the basic single-pollutant models were used and the models were as follows:

log[*E*(*Y_t_*)] = *βZ_t_* + *ns* (*time,* 7 x 5) + *ns*(*Temp_t_*, 3) + *ns*(*RH_t_*, 3) + *DOW* + *Holiday_t_* + *intercept*

*LOS_t_* = *βZ_t_* + *ns*(*time*, 7 x 5) + *ns*(*Temp_t_*, 3) + *ns*(*RH_t_*, 3) + *DOW* + *Holiday_t_* + *intercept*

*Cost_t_* = *βZ_t_* + *ns*(*time*, 7 + 5) + *ns*(*Temp_t_*, 3) + *ns*(*RH_t_*, 3) + *DOW* + *Holiday_t_* + *CPI* + *intercept*

where *E(Y_t_)* represents the expected daily RD-MD counts on day *t*; *LOS_t_* is the length of hospital stay on day *t*; *Cost_t_* is hospital cost on day *t*; β is the relative coefficient; *Z_t_* indicates the concentration of air pollutants on day *t*; *ns* represents natural smooth splined function; *time* is the days of calendar time on day *t*, used to control the long-term trend and seasonality of time; *Temp_t_* refers to the temperature on day *t*; *RH_t_* indicates the relative humidity on day *t*; *DOW* is day of the week, *CPI* is consumer price index.

The association between air pollution and outcome was expressed as percentage changes (%, PC) and the corresponding 95% confidence interval (CI) for hospital admissions and absolute increase and the corresponding 95% CI for LOS and hospital cost, respectively [16,36]. The calculation formula is below:

*PC* = [exp(β x *Δc* - 1] x 100%

Absolute increase = β x Δc

where β represents coefficient of air pollutants from GAM model; *Δc* is the unit increase number of air pollutants concentration. For PM_2.5_, PM_10_, SO_2_, NO_2_, O_3_, we set *Δc* as 10 μg/m^3^; for CO, we set *Δc* as 1 milligramme per cubic metre (mg/m^3^).

#### Estimating the attributable risk

Attributable fraction (AF) and attributable number (AN) based on the previously established GAM model were used to estimate the burden of RD-DM due to exceeding air pollutants exposure. The calculation formula is below:



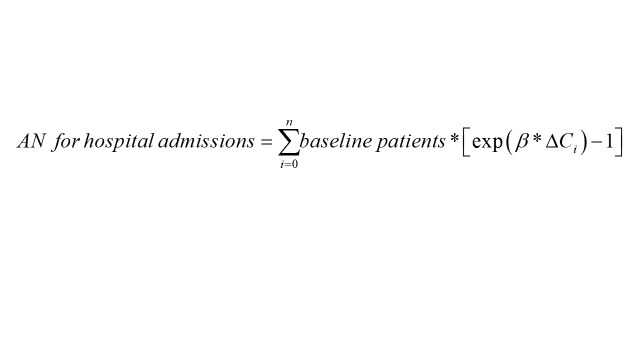





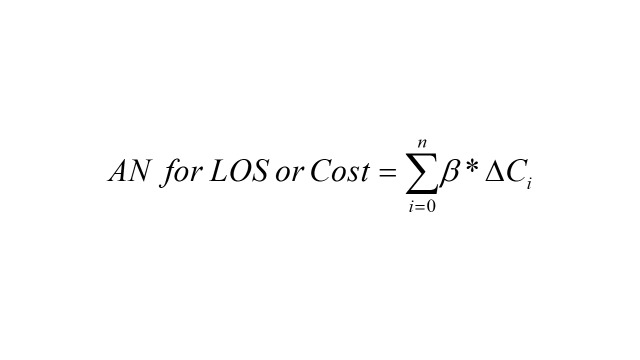





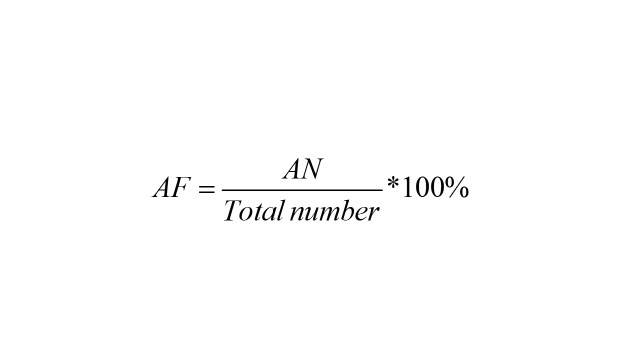



where baseline patients are the admissions number on one day; *i* represents days when air pollution concentration is higher than the reference concentration; β is the exposure–response relation coefficient based on the GAM; *∆C_i_* stands for the difference between the daily concentration of air pollutants and the reference concentration. We choose different standards as reference concentrations: 1) the standard of WHO’s 2021 AQG [25]; 2) WHO reports that there is no threshold concentration for adverse health effects of six air pollutants [25,37]. Therefore, we also selected 0 μg/m^3^ as reference concentration.

#### Exposure-response relationship

We used the *plot.gam()* function from *mgcv* package to explore nonlinear relationship. Since the link function of the model must be a log function, we only examined the nonlinear relationship between air pollution and hospital admissions.

#### Subgroup analyses

Furthermore, stratified subgroup analysis by age (<65 vs. ≥65) and sex (male vs. female) was utilised to examine differences in effect values. And Z-test was employed to test the statistical significance of the differences between subgroups. The equation was listed below:



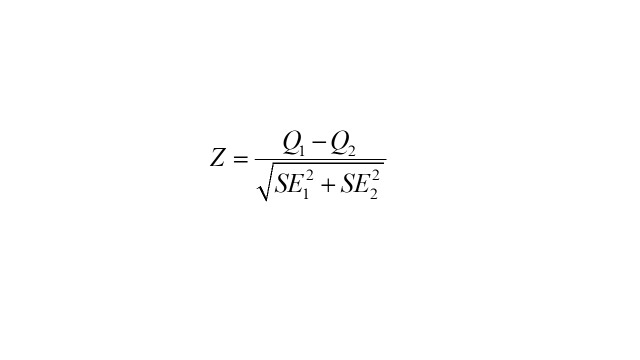



where *Q_1_* and *Q_2_* refer to the effect values of the subgroups, respectively; SE*_1_* and SE*_2_* refer to the standard error of the subgroups, respectively [38].

#### Sensitivity analysis

For the purpose of ensuring that our results are robust, we conducted the following sensitivity analyses: 1) to test the stability of the effects after other pollutants are taken into account, two-pollutant models were built; PM_10_ and PM_2.5_ do not appear simultaneously in the model considering the collinearity between the two air pollutants; 2) we changed the *df* of the long-term trend from 4 to 12 *df* per year; 3) E-value was computed to assess the unmeasured confounding bias. The results are considered robust if the risk ratio (RR) is less than the corresponding E-value [16,39,40]; 4) we used PM_10_ and PM_2.5_ from China High Air Pollutants (CHAP) data (http://www.geodata.cn), a data derived from model, to compare the results of fixed monitoring stations data with that of the model-based data.

All statistical analyses were performed using R software (version 4.2.1), and a two-sided *P* < 0.05 was considered statistically significant.

## RESULTS

### Results summary

Our results suggest that daily PM_2.5_, PM_10_, SO_2_, NO_2_, and CO contributed significantly to elevated risk of hospital admissions, LOS, and hospital cost for patients with RD-DM. Overall, 32.68% of hospital admissions for RD-DM could be attributed to NO_2_, with the highest population attributable faction. O_3_ showed protective effects on hospital admissions, LOS, and hospital cost among RD-DM patients, though two-pollutant models suggest the potential joint risk effects of O_3_ with other pollutants. The effects of air pollutants on hospital admissions, LOS and economic cost for RD-DM were more pronounced in people over the age of 65 and male patients than their counterparts.

### Baseline characteristics

We included 17 320 RD-MD patients in our study (Figure S1 in the **Online Supplementary Document**), leading to 213 082 days in LOS and 207.50 million CNY in hospital costs. Daily RD-DM admissions, LOS, and hospital costs ranged from five to 29 cases, 0.00 to 676.00 days, and 0.00 to 712 021.87 CNY, respectively (**Table 1**). A total of 62.74% of the patients were over 65 years old. There were 10 785 (62.27%) male patients and 6535 (37.33%) female patients (Table S1 in the **Online Supplementary Document**). Daily mean concentrations were 28.24 μg/m^3^ for PM_2.5_, 51.88 μg/m^3^ for PM_10_, 32.05 μg/m^3^ for SO_2_, 33.54 μg/m^3^ for NO_2_, 1.39 mg/m^3^ for CO, 80.85 μg/m^3^ for O_3_; in terms of daily mean temperatures and relative humidity, they were 21.02°C and 58.73%, respectively (**Table 1**). Figure S2 in the **Online Supplementary Document** shows correlations between air pollution levels and meteorological variables. PM_2.5_ and PM_10_ showed the strongest correlation. The calendar heat map (Figure S3 in the **Online Supplementary Document**) shows the daily variation in hospital admissions, LOS and hospital cost for participants.

**Table 1:** Distribution characteristics of daily cases of diabetes mellitus and comorbid respiratory diseases (RD-DM), air pollutants and meteorological factors in Panzhihua from 2016 to 2020

Variables	Mean	SD	Min	P_25_	P_50_	P_75_	Max
Admissions	9.48	5.00	0.00	6.00	9.00	12.00	29.00
LOS (days)	116.63	69.90	0.00	67.00	105.00	153.00	676.00
Hospital cost (Yuan CNY)	113 578.50	80 110.29	0.00	61 088.45	94 482.18	148 171.64	712 021.87
PM_2.5_ (μg/m^3^)	28.24	11.67	7.00	20.00	27.00	34.00	134.00
PM_10_ (μg/m^3^)	51.88	18.31	14.00	38.00	50.00	63.00	157.00
SO_2_ (μg/m^3^)	32.05	13.29	9.00	23.00	30.00	38.00	137.00
NO_2_ (μg/m^3^)	33.54	10.23	14.00	26.00	31.00	40.00	73.00
CO (mg/m^3^)	1.39	0.50	0.50	1.10	1.30	1.60	6.00
O_3_ (μg/m^3^)	80.85	29.95	14.00	59.00	77.00	100.50	196.00
Temperature (°C)	21.02	5.45	5.03	16.30	21.83	25.23	33.55
Relative humidity (%)	58.73	18.34	14.00	43.89	62.75	72.75	96.83

SD – standard deviation, Min – minimum value, P_25_ – first quantile, P_50_ – second quantile, P_75_ – third quantile, Max – maximum value, LOS – length of hospital stay, PM_2.5_ – particles with an aerodynamic diameter <2.5 micron (μm), ug/m^3^ – microgrammes per cubic metre, PM_10_ – particles with an aerodynamic diameter <10 μm, SO_2_ – sulfur dioxide, NO_2_ – nitrogen dioxide, CO – carbon monoxide, mg/m^3^ – milligrammes per cubic metre, O_3_ – ozone

### Effect of air pollution on RD-DM

**Figure 1:**
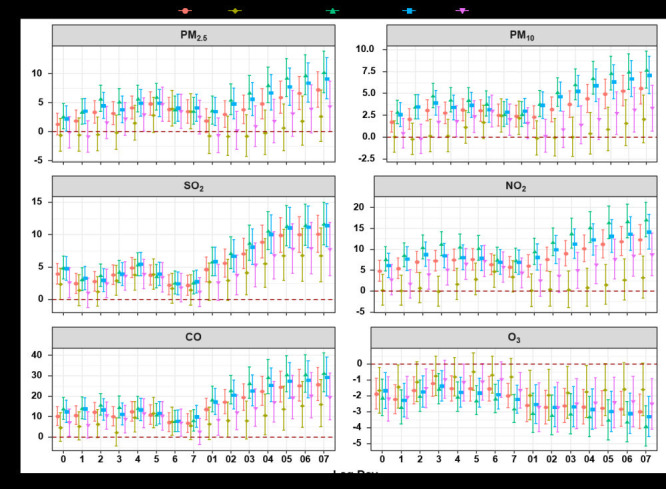
The effect of six air pollutants on admission with single pollutant model. Percentage change for particles with an aerodynamic diameter <2.5 micron (μm) (PM_2.5_), particles with an aerodynamic diameter <10 μm (PM_10_), sulfur dioxide (SO_2_), nitrogen dioxide (NO_2_) and ozone (O_3_) were per 10 µgmes per cubic metre (ug/m^3^) increase and 1 mgmes per cubic metre (mg/m^3^) for carbon monoxide (CO); younger adult: age less than or equal to 65 years old; the elderly: age over 65 years old.

**Figure 1** illustrates the PC value for six air pollutants with varying lag days in RD-DM hospital admissions. All air pollutants except O_3_ show statistically adverse effects. There was a maximum effect value at lag 07 for six pollutants, with PM_2.5_ being 7.25% (95% CI = 4.26 to 10.33), PM_10_ being 5.59% (95% CI = 3.79 to 7.42), SO_2_ being 10.10% (95% CI = 7.29 to 12.98), NO_2_ being 12.33% (95% CI = 8.82 to 15.95), CO being 25.77% (95% CI = 17.88 to 34.19), and O_3_ being -2.99% (95% CI = -4.08 to -1.90). Subgroup analysis shows that the effect is more pronounced in people over 65 than in young adults; similarly, the effect is stronger in male patients than in female patients (Table S2 to Table S13 in the **Online Supplementary Document**).

**Figure 2:**
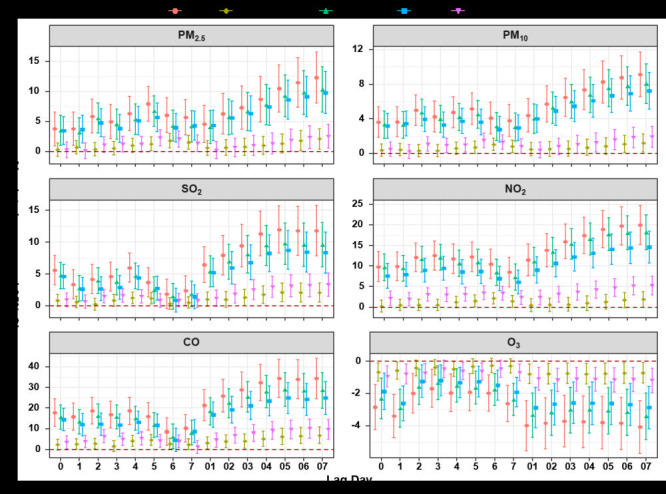
The effect of six air pollutants on length of hospital stay (LOS) with single pollutant model. Absolute increase for particles with an aerodynamic diameter <2.5 micron (μm) (PM_2.5_), particles with an aerodynamic diameter <10 μm (PM_10_), sulfur dioxide (SO_2_), nitrogen dioxide (NO_2_) and ozone (O_3_) were per 10 µgmes per cubic metre (ug/m^3^) increase and 1 mgmes per cubic metre (mg/m^3^) for carbon monoxide (CO); younger adult: age less than or equal to 65 years old; the elderly: age over 65 years old.

**Figure 2** shows the absolute increase with LOS in six air pollutants with different lag days. All air pollutants except O_3_ show statistically different adverse effects. For LOS, the lag day of maximum effect value slightly varies among six pollutants for the total population in which PM_2.5_ is 12.33 days (95% CI = 8.06 to 16.61) at lag 07, PM_10_ is 9.15 days (95% CI = 6.56 to 11.74) at lag 07, SO_2_ is 11.98 days (95% CI = 8.29 to 15.67) at lag 05, NO_2_ is 20.00 days (95% CI = 15.24 to 24.76) at lag 07, CO is 34.37 days (95% CI = 24.58 to 44.17) at lag 07 and O_3_ is -4.07 days (95% CI = -5.68 to -2.46) at lag 07. In age subgroup analysis, the effect is more pronounced in people over the age of 65 than in the young adult; similarly, the effect is stronger in male patients than in female patients (Table S2 to Table S13 in the **Online Supplementary Document**).

**Figure 3:**
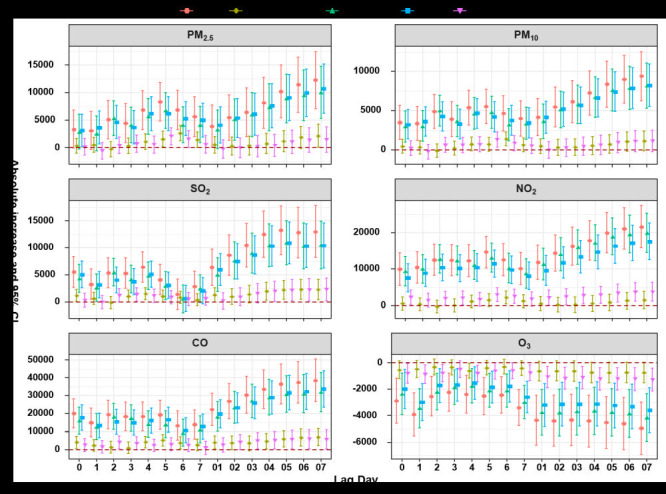
The effect of six air pollutants on hospital cost with single pollutant model. Absolute increase for particles with an aerodynamic diameter <2.5 micron (μm) (PM_2.5_), particles with an aerodynamic diameter <10 μm (PM_10_), sulfur dioxide (SO_2_), nitrogen dioxide (NO_2_) and ozone (O_3_) were per 10 µgmes per cubic metre (ug/m^3^) increase and 1 mgmes per cubic metre (mg/m^3^) for carbon monoxide (CO); younger adult: age less than or equal to 65 years old; the elderly: age over 65 years old.

**Figure 3** shows the absolute increase with hospital cost in six air pollutants with different lag days. All air pollutants except O_3_ show statistically different adverse effects. The lag day of maximum effect value slightly varies among six pollutants for total population in which PM_2.5_ is 12 279.18 CNY (95% CI = 7063.50 to 17 494.86) at lag 07, PM_10_ is 9397.81 CNY (95% CI = 6234.08 to 12 561.53) at lag 07, SO_2_ is 13 269.71 CNY (95% CI = 8771.65 to 17 767.76) at lag 05, NO_2_ is 21 551.46 CNY (95% CI = 15 750.94 to 27 351.97) at lag 07, CO is 38 540.25 CNY (95% CI = 26 593.88 to 50 486.62) at lag 07 and O_3_ is -4942.53 CNY (95% CI = -6906.63 to -2978.44) at lag 07. Subgroup analysis shows the effect is more pronounced in people over the age of 65 than in the young adult; similarly, the effect is stronger in male patients than in female patients (Table S2 to Table S13 in the **Online Supplementary Document**).

### Attributable risk of RD-DM due to air pollution

**Table 2** summarised the attributable risk due to exceeding air pollutants exposure using the standard of WHO’s 2021 AQG and 0 μg/m^3^ as the reference concentrations, respectively. For hospital admissions in the total population, the maximum AF in six pollutants is NO_2_ using 0 μg/m^3^ as the reference concentration which we estimated that about 32.68% (95% CI = 25.12% to 39.42%) corresponding to an AN of 5661 (95% CI = 4351 to 6827) RD-DM patients. In subgroup populations, we found similar results (Table S17 and Table S18 in the **Online Supplementary Document**). In terms of LOS and hospital cost, PM_2.5_, PM_10_, SO_2_, NO_2_ and O_3_ will significantly reduce more LOS and hospital cost using 0 μg/m^3^ as the reference, compared to using the standard of WHO’s 2021 AQG as the reference. And the AF of NO_2_ is highest. Gender-specific subgroup analyses found similar results (Table S19 and Table S21 in the **Online Supplementary Document**). However, age-specific subgroup analyses found different results (Table S20 and Table S22 in the **Online Supplementary Document**).

**Table 2:** The attributable risk for different population with different air quality guideline

Type	Air Pollution	Guideline	Attributable number	Attributable fraction (%)
**Admissions**				
WHO 2021	PM_2.5_	15	1605 (987-2185)	9.27 (5.70-12.61)
	PM_10_	45	1063 (746-1365)	6.14 (4.30-7.88)
	SO_2_	40	424 (319-523)	2.45 (1.84-3.02)
	NO_2_	25	1847 (1384-2283)	10.67 (7.99-13.18)
	CO	4	10 (7-12)	0.06 (0.04-0.07)
Threshold concentration = 0	PM_2.5_	0	3144 (1960-4223)	18.15 (11.32-24.38)
	PM_10_	0	4308 (3091-5409)	24.87 (17.84-31.23)
	SO_2_	0	4606 (3527-5589)	26.60 (20.37-32.27)
	NO_2_	0	5661 (4351-6827)	32.68 (25.12-39.42)
	CO	0	4793 (3611-5860)	27.67 (20.85-33.84)
**LOS**				
WHO 2021	PM_2.5_	15	30 453 (19 901-41 006)	14.29 (9.34-19.24)
	PM_10_	45	18 220 (13 058-23 381)	8.55 (6.13-10.97)
	SO_2_	40	5300 (3531-7070)	2.49 (1.66-3.32)
	NO_2_	25	33 607 (25 610-41 605)	15.77 (12.02-19.53)
	CO	4	69 (49-88)	0.03 (0.02-0.04)
Threshold concentration = 0	PM_2.5_	0	63 631 (41 581-85 680)	29.86 (19.51-40.21)
	PM_10_	0	86 683 (62 126-111 240)	40.68 (29.16-52.21)
	SO_2_	0	69 249 (46 128-92 370)	32.5 (21.65-43.35)
	NO_2_	0	122 577 (93 407-151 747)	57.53 (43.84-71.22)
	CO	0	87 406 (62 503-112 309)	41.02 (29.33-52.71)
**Hospital cost**				
WHO 2021	PM_2.5_	15	30 319 751 (17 441 186-43 198 317)	14.61 (8.41-20.82)
	PM_10_	45	18 721 375 (12 418 920-25 023 830)	9.02 (5.98-12.06)
	SO_2_	40	5 820 466 (3 665 383-7 975 548)	2.80 (1.77-3.84)
	NO_2_	25	36 210 758 (26 464 736-45 956 781)	17.45(12.75-22.15)
	CO	4	77 080 (53 188-100 973)	0.04 (0.03-0.05)
Threshold concentration = 0	PM_2.5_	0	63 351 973 (36 442 698-90 261 249)	30.53(17.56-43.50)
	PM_10_	0	89 069 613 (59 084 785-119 054 441)	42.92 (28.47-57.37)
	SO_2_	0	76 047 935 (47 890 462-104 205 407)	36.65 (23.08-50.22)
	NO_2_	0	132 073 796 (96 526 511-167 621 080)	63.65(46.52-80.78)
	CO	0	98 000 147 (67 622 919-128 377 374)	47.23(32.59-61.87)

PM_2.5_ – particles with an aerodynamic diameter <2.5 micron (μm), PM_10_ – particles with an aerodynamic diameter <10 μm, SO_2_ – sulfur dioxide, NO_2_ – nitrogen dioxide, CO – carbon monoxide, LOS – length of hospital stay

### The nonlinear relationship between air pollution and hospital admissions

Figure S10 in the **Online Supplementary Document** shows the exposure-response relationship for six air pollutants and hospital admissions. Each air pollutant exhibited approximately U-shaped curves with different degrees of curvature, indicating that air pollution affects hospital admissions in a nonlinear way. For example, the slopes are flat at low PM_10_ concentrations, while the slopes are steeper at 50 μg/m^3^ and higher concentrations.

### Sensitivity analysis

The results of two-pollutant models at lag 07-day were showed in **Table 3**. After adjustment for other air pollutants, the effect estimates were still statistically significant. Table S14 to Table S16 in the **Online Supplementary Document** showed the results of two-pollutant models along different single-day lag structures. Figure S4 to Figure S6 in the **Online Supplementary Document** showed the results based on CHAP data were largely consistent with results based on fixed monitoring stations. Additionally, our results remained relatively stable after *df* of time was adjusted (4-12 *df* per year) (Figure S7 to Figure S9 in the **Online Supplementary Document**). And almost all of the E-values calculated for the total population along different lag structures exceed the original RR (Table S23 in the **Online Supplementary Document**).

**Table 3:** The effect of six air pollutants on admissions, length of hospital stay (LOS), and hospital cost for diabetes mellitus and comorbid respiratory diseases (RD-MD) associated with one unit increase in six air pollutants at lag 07 days using single- and two-pollutant models

	PM_2.5_	PM_10_	SO_2_	NO_2_	CO	O_3_
**Admissions**						
None	7.25 (4.26-10.33)*	5.59 (3.79-7.42)*	10.10 (7.29-12.98)*	12.33 (8.82-15.95)*	25.77 (17.88-34.19)*	-2.99 (-4.08,-1.90)*
***+*** *PM_2.5_*	-	-	10.12 (7.26-13.06)*	12.99 (9.26-16.85)*	25.93 (17.83-34.60)*	-3.24 (-4.34,-2.13)*
*+PM* ***_10_***	-	-	9.69 (6.78-12.68)*	12.65 (8.69-16.76)*	24.56 (16.34-33.36)*	-3.14 (-4.23,-2.04)*
*+SO* ***_2_***	6.10 (3.08-9.20)*	4.77 (2.93-6.64)*	-	11.10 (7.58-14.73)*	21.84 (13.90-30.33)*	-2.78 (-3.87,-1.68)*
*+NO* ***_2_***	5.88 (2.64-9.22)*	5.04 (3.01-7.12)*	9.38 (6.53-12.32)*	-	23.53 (15.03-32.65)*	-2.87 (-3.96,-1.76)*
***+*** *CO*	5.61 (2.48-8.84)*	4.73 (2.81-6.67)*	9.02 (6.13-11.99)*	10.96 (7.10-14.97)*	-	-2.73 (-3.83,-1.63)*
*+O* ***_3_***	8.93 (5.84-12.11)*	6.03 (4.23-7.87)*	9.51 (6.72-12.38)*	11.56 (8.10-15.12)*	23.48 (15.72-31.75)*	-
**LOS**						
None	12.33 (8.06-16.61)*	9.15 (6.56-11.74)*	11.83 (7.88-15.78)*	20.00 (15.24-24.76)*	34.37 (24.58-44.17)*	-4.07 (-5.68,-2.46)*
*+PM_2.5_*	-	-	11.19 (7.15-15.23)*	20.15 (15.08-25.22)*	33.19 (23.06-43.33)*	-4.63 (-6.26,-3.00)*
*+PM* ***_10_***	-	-	10.32 (6.21-14.43)*	19.62 (14.25-24.98)*	31.33 (20.94-41.72)*	-4.35 (-5.95,-2.74)*
*+SO* ***_2_***	10.81 (6.48-15.14)*	8.12 (5.47-10.78)*	-	18.72 (13.87-23.56)*	30.45 (20.28-40.63)*	-3.81 (-5.41,-2.20)*
*+NO* ***_2_***	9.23 (4.58-13.87)*	7.53 (4.62-10.45)*	9.98 (5.95-14.01)*	-	28.60 (17.72-39.48)*	-3.80 (-5.41,-2.20)*
*+CO*	9.66 (5.15-14.16)*	7.70 (4.96-10.44)*	9.66 (5.57-13.75)*	17.85 (12.56-23.14)*	-	-3.64 (-5.25,-2.03)*
*+O* ***_3_***	15.14 (10.78-19.50)*	10.12 (7.52-12.73)*	11.24 (7.29-15.19)*	19.53 (14.80-24.26)*	32.68 (22.86-42.5)*	–
**Hospital cost**						
None	12 279.18 (7063.50-17 494.86)*	9397.81 (6234.08-12 561.53)*	12 989.21 (8179.83-17 798.59)*	21 551.46 (15 750.94-27 351.97)*	38 540.25 (26 593.88-50 486.62)*	-4942.53 (-6906.63,-29 78.44)*
*+PM_2.5_*	-	-	12 517.65 (7596.19-17 439.11)*	22 160.09 (15 980.06-28 340.11)*	37 903.73 (25 549.43-50 258.03)*	-5513.15 (-7506.08,-3520.21)*
*+PM* ***_10_***	-	-	11 693.38 (6687.84-16 698.92)*	21 865.10 (15 318.17-28 412.02)*	36 018.08 (23 389.19-48 646.97)*	-5228.95 (-7191.21,-3266.69)*
*+SO* ***_2_***	10 790.18 (5497.26-16 083.10)*	8425.30 (5176.32-11 674.29)*	-	20 177.81 (14 286.23-26 069.39)*	34 677.18 (22 312.26-47 042.11)*	-4702.19 (-6665.55,-2738.83)*
*+NO* ***_2_***	9098.34 (3417.15-14 779.54)*	7814.81 (4255.03-11 374.59)*	11 148.55 (6234.41-16 062.68)*	-	33 137.94 (19 927.70-46 348.18)*	-4694.91 (-6654.27,-2735.54)*
***+*** *CO*	9064.15 (3567.13-14 561.17)*	7658.95 (4316.36-11 001.55)*	10 498.20 (5526.74-15 469.67)*	18 904.98 (12 463.38-25 346.58)*	-	-4475.88 (-6441.94,-2509.82)*
*+O* ***_3_***	14 740.09 (9398.64-20 081.55)*	10 197.56 (7006.65-13 388.47)*	12 216.32 (7376.74-17 055.91)*	21 194.78 (15 398.88-26 990.68)*	36 990.83 (24 996.11-48 985.54)*	-

PM_2.5_ – particles with an aerodynamic diameter <2.5 micron (μm), PM_10_ – particles with an aerodynamic diameter <10 μm, SO_2_ – sulfur dioxide, NO_2_ – nitrogen dioxide, CO – carbon monoxide, O_3_ – ozone, LOS – length of hospital stay

*P < 0.05.

## DISCUSSION

The relationship between air pollution and RD or DM has been estimated in previous studies. To our knowledge, this is one of the few studies to investigate the hospital admissions, LOS, and hospital cost among patients with RD-DM due to air pollution. Our results suggest that daily PM_2.5_, PM_10_, SO_2_, NO_2_, and CO contributed significantly to elevated risk of hospital admissions, LOS, and hospital cost for patients with RD-DM. Overall, 32.68% of hospital admissions for RD-DM could be attributed to NO_2_, with the highest population attributable faction. O_3_ showed protective effects on hospital admissions, LOS, and hospital cost among RD-DM patients, though two-pollutant models suggest the potential joint risk effects of O_3_ with other pollutants. The effects of air pollutants on hospital admissions, LOS and economic cost for RD-DM were more pronounced in people over the age of 65 and male patients than their counterparts.

The effects of most air pollutants on hospital admissions for RD-DM in lag 0 were relatively low and gradually reached a maximum effect at lag 07 in this study. A previous study focusing on only RD or DM suggested different trends. For example, a study of 234 279 patients hospitalised due to RD suggested that the effects of all pollutants except SO_2_ were highest in lag 0 while there was a gradual decrease in the cumulative effects of PM_2.5_ and NO_2_ [41]. A study of 88 904 patients with DM suggested an increased risk of hospital admissions for DM at lag 0-2 days [33]. Moreover, our result suggested that increasing PM_2.5_ by 10 mg/m^3^ could increase hospital admission risk for RD-DM in lag 2 by 3.38% (95% CI = 1.45 to 5.34%). A previous study suggested an increase of 10 ug/m^3^ in PM_2.5_ could increase the risk of hospital admissions for DM at lag 0-2 days by 1.71% (95% CI = 0.56 to 2.87%) [33], which is lower than the effect observed in our study. A meta-analysis also suggested that PM_2.5_ had a greater effect size in patients with RD-DM than those only with RD [42].

There are some possible explanations for the observed stronger effect of air pollutants on hospital admissions for patients with RD-DM. First, for individuals with comorbid DR and DM, disorders in both the respiratory and endocrine systems may grant them poorer immune responses and make them more susceptible to air pollutants. Previous studies have suggested that chronic hyperglycaemia can affect innate immune system acting on chemotaxis, phagocytosis and bactericidal activity of neutrophils and macrophages [43]. In animal models, inflammation, lung parenchyma, and vascular impairment are all associated with hyperglycaemia, and high levels of glucose in the airways can promote pathogen growth, decreased lung function [4,44]. Second, our study was conducted in a site with typical DHVs climate. The difference of climate conditions may affect the disease incidence [45]. Our study area has a low level of air pollution, e.g. the median value of SO_2_ (30.0 ug/m^3^) is lower than the criteria of WHO (40.0 ug/m^3^ for SO_2_), yet the strong effect of air pollutants on hospital admissions for RD-DM confirmed the previous conclusions that the health vulnerability of populations in areas with low air pollution exposure level is still noteworthy [46]. Third, heterogeneity in chemical components of air pollutants and demographic composition (e.g. a higher level of ageing in Panzhihua than the average level in China (15.9 vs. 14.9%) [47]) might also account for the different health effects [48].

Intriguingly, our study and sensitive analysis with other data revealed a negative association between O_3_ and hospital admissions for RD-DM, which is consistent with previous studies [49,50]. A potential explanation is that O_3_ is negatively associated with PM levels and thus the protective effect is merely an artifact of the negative association [49]. As our multiple pollutant models suggested, O_3_ combined with other pollutants, especially PM_2.5_, would increase the risk of hospital admissions for RD-DM. Another potential explanation is that the levels of O_3_ in our study site are lower than the Chinese National Ambient Air Quality Standard [51], which is in a range that has a protective effect on health. Since inconsistent evidence also exists [52], more studies are still needed to clarify the effect of O_3_.

Significant associations between air pollutants and LOS and hospital cost for RD-DM were observed in our study. There are some similarities and differences in the effects of specific pollutants compared to current studies. First, similar to Li Zhiwei’s study [19], our study suggested that PM will significantly increase LOS and hospital cost due to DM being comorbid with other diseases. A previous study in United States suggested that hospital cost increased by 47 dollars when monthly PM_2.5_ increased by 1 mg/m^3^ [53]. Second, our study suggested that the lag day of maximum effect value for LOS and hospital cost due to NO_2_ was 20.00 days (95% CI = 15.24 to 24.76) and 21 551.46 CNY (95% CI = 15 750.94 to 27 351.97) at lag 07. However, a previous study in China found the rise in 16-day average NO_2_ by 10-μg/m^3^ was correlated with negative alterations in LOS and hospital cost for patients with T2D [54]. Third, we found the LOS attributable fraction of NO_2_ is highest in all pollutants and subgroups, which is inconsistent to the previous study focusing on CVD-DM which suggests PM_2.5_ had the maximum attributable fraction [19]. As a common urban pollutant and precursor of PM and O_3_, our findings indicated that the effect of NO_2_ on LOS for respiratory system could be already notable before it alters to other chemical components.

We found some vulnerable subpopulation under the impacts of air pollution. Specifically, the effect of air pollution on participants >65 years old and male were slightly higher than their counterparts. This might be explained by poorer immune and metabolism function, and worse health condition in the elderly due to ageing and other comorbidities [12,55]. Besides, previous environmental epidemiological studies suggested that males are vulnerable to air pollution [13,35] (though exception exists [49]), due to males tend to have unhealthy lifestyles and spend more time outdoor than females, which may make them more likely to inhale pollutants [56]. For those who are frequently hospitalised due to poor condition, conceivably, their LOS and hospital cost would consequently increase, which might explain why we observe a similar trend of air pollutants’ effects on hospital admissions, LOS, and hospital cost in the total population and different subgroups.

Some potential mechanisms should be declared about how these pollutants affect the health of RD-DM patients. First, exposure to air pollutants may lead to the release of cytokines and inflammatory factors, which could further induce lung inflammation, reduce insulin sensitivity, and block the uptake of glucose in peripheral tissues [57]. Second, air pollutants can elicit inflammation that may lead to the risk of metabolic syndrome including diabetes, hypertension, and obesity [58]. Third, air pollutants may could affect the gastrointestinal tract, and may further affect the function of gut microbiota to affect the prognosis of DM [59].

### Limitations

Some limitations should be mentioned. First, we fail to capture the dynamic movement of individuals with the assumption that all participants were with the same levels of ambient air pollutants exposure based on fixed monitoring stations, which may result in measurement errors. Second, the focus of our study was only on patients with total RD comorbid and total DM, and may not all subtypes of these two diseases are related to air pollution. Third, we fail to consider the interaction between variables since GAM has an inherent limitation in restricting the impact of various interactions. Fourth, the absence of some survey data, such as other environmental factors (e.g. noise, green space) and lifestyle habits (e.g. physical activity, passive smoking, diet), may limit the accuracy of the results. Further studies taking into account these potential confounders are needed to verify the relationship between air pollution and comorbidities. Fifth, we cannot draw causal inferences since this is an observational study and some important confounding factors may fail to consider. More studies that include the more information abovementioned can help strengthen our findings in the future. Sixth, due to limited ambient air pollution data availability, it was impossible to adjust all the ambient air pollution, such as Volatile Organic Compounds (VOCs), Nitrogen Oxides (NOx), dust and smoke.

## CONCLUSIONS

This study found that most ambient air pollutants (PM_2.5_, PM_10_, SO_2_, NO_2_, and CO) were associated with increased risk of hospital admissions, LOS, and hospital cost for patients with RD-DM, and the effects were pronounced in people over the age of 65 and male patients. Our study complements the evidence on the relationship between ambient air pollutants and comorbidity from multi-dimensional measurement of risk, hospitalisation time, and economic cost in low-pollution areas of LMICs. Policy changes to reduce air pollutants exposure may lead to improved RD-DM admissions and substantial savings in health care spending and hospital stay.

## Additional material


Online Supplementary Document
