# Supplementary material for: The impact of ambient air pollution on hospital admissions, length of stay and hospital costs for patients with diabetes mellitus and comorbid respiratory diseases in Panzhihua, Southwest China
Source: J Glob Health. 2023 Oct 13;13:04118. doi: 10.7189/jogh.13.04118 (PMC10570759; doi:10.7189/jogh.13.04118)
Supplement: Online Supplementary Document [file jogh-13-04118-s001.pdf]

Supplementary material

The impact of ambient air pollution on hospital admissions, length of stay, and hospital costs for patients with diabetes mellitus and comorbid respiratory diseases in Panzhihua, Southwest China

Xianzhi Li[1,2,3†], Bin Yu[4†], Yajie Li[5†], Haorong Meng[6], Meiying Shen[7], Yan Yang[1,3,8], Zonglei Zhou[9], Shunjin Liu[1,2,3], Yunyun Tian[2,3], Xiangyi Xing[1,3,10]*, Li Yin[1,2,3]*

[1]Meteorological Medical Research Center, Panzhihua Central Hospital, Panzhihua, China
[2]Clinical Medical Research Center, Panzhihua Central Hospital, Panzhihua, China
[3]Dali University, Dali, China
[4]Institute for Disaster Management and Reconstruction, Sichuan University - Hong Kong Polytechnic University, Chengdu, China
[5]Tibet Center for Disease Control and Prevention, Lhasa, China
[6]Yunnan Center for Disease Control and Prevention, Kunming, China
[7]Nursing department, Panzhihua Central Hospital, Panzhihua, China
[8]Department of Respiratory and Critical Care Medicine, Panzhihua Central Hospital, Panzhihua, China
[9]Department of Epidemiology, School of Public Health, Fudan University, Shanghai, China
[10]Department of Pharmacy, Panzhihua Central Hospital, Panzhihua, China

[†]These authors contributed equally to this work.

*Address correspondence to:
Dr. Xiangyi Xing
No.34, Yikang Street, East District, Panzhihua, 617067, China
E-mail addresses: xianzhi_scu@163.com

Dr. Li Yin
No.34, Yikang Street, East District, Panzhihua, 617067, China
E-mail addresses: 425281415@qq.com

Contents

Supplementary material: Statistical Analyses Plan 1

Supplementary material: Figures 3

Figure S1 Flow chart of this study.. 3

Figure S2 Spearman correlations of air pollutants and meteorological factors in Panzhihua from 2016 to 2020. 4

Figure S3 Calendar heat map of daily admissions, LOS, hospital cost for RD-DM in Panzhihua from January 1, 2016 to December 31, 2020. 5

Figure S4 The effect of air pollutants on hospital admissions with single pollutant model for total population using different air pollution data. 6

Figure S5 The effect of air pollutants on length of hospital stay (LOS) with single pollutant model for total population using different air pollution data. 7

Figure S6 The effect of air pollutants on hospital cost with single pollutant model for total population using different air pollution data. 8

Figure S7 Percentage changes for RD-DM admissions with different degrees of freedom of time trends at lag 07 day. 9

Figure S8 Absolute increase in LOS (length of hospital stay) for RD-DM with different degrees of freedom of time trends at lag 07 day. 10

Figure S9 Absolute increase in hospital cost for RD-DM with different degrees of freedom of time trends at lag 07 day. 11

Figure S10 Dose-response relationship between admissions and air pollutants. 12

Supplementary material: Tables 13

Table S1 Demographic characteristics of different subgroups of the population 13

Table S2 The $PM_{2.5}$ effect of admissions, LOS and hospital cost for RD-DM on gender group with different lag day. 14

Table S3 The $PM_{2.5}$ effect of admissions, LOS and hospital cost for RD-DM on age group with different lag day. 15

Table S4 The PM_{10} effect of admissions, LOS and hospital cost for RD-DM on gender group with different lag day. 16

Table S5 The PM_{10} effect of admissions, LOS and hospital cost for RD-DM on age group with different lag day. 17

Table S6 The SO_2 effect of admissions, LOS and hospital cost for RD-DM on gender group with different lag day. 18

Table S7 The SO_2 effect of admissions, LOS and hospital cost for RD-DM on age group with different lag day. 19

Table S8 The NO_2 effect of admissions, LOS and hospital cost for RD-DM on gender group with different lag day. 20

Table S9 The NO_2 effect of admissions, LOS and hospital cost for RD-DM on age group with different lag day. 21

Table S10 The CO effect of admissions, LOS and hospital cost for RD-DM on gender group with different lag day. 22

Table S11 The CO effect of admissions, LOS and hospital cost for RD-DM on age group with different lag day. 23

Table S12 The O_3 effect of admissions, LOS and hospital cost for RD-DM on gender group with different lag day. 24

Table S13 The O_3 effect of admissions, LOS and hospital cost for RD-DM on age group with different lag day. 25

Table S14 Percent change (95% CI）in hospital admissions for RD-DM associated with one unit increase in $PM_{2.5}$, PM_{10}, SO_2, NO_2, CO and O_3 along different lag days using single-pollutant and two-pollutant models. 26

Table S15 Absolute increase (95% CI） in LOS (length of hospital stay) for RD-DM associated with one unit increase in $PM_{2.5}$, PM_{10}, SO_2, NO_2, CO and O_3 along different lag days using single-pollutant and two-pollutant models. 28

Table S16 Absolute increase (95% CI） in hospital cost for RD-DM associated with one unit increase in $PM_{2.5}$, PM_{10}, SO_2, NO_2, CO and O_3 along different lag days using single-pollutant and two-pollutant models. 30

Table S17 The admissions attributable risk for different population with different air quality guideline by gender group. 33

Table S18 The admissions attributable risk for different population with different air quality guideline by age group. 34

Table S19 The LOS attributable risk for different population with different air quality guideline by gender group. 35

Table S20 The LOS attributable risk for different population with different air quality guideline by age group. 36

Table S21 The hospital cost attributable risk for different population with different air quality guideline by gender group. 36

Table S22 The hospital cost attributable risk for different population with different air quality guideline by age group. 38

Table S23 Results of E value analysis for association between air pollutants with RD-DM admissions in different lag days. 39

Table S24 Time series data example for RD-DM patients 41

Supplementary material: Statistical Analyses Plan

16 September 2022

1. Objective

To assess the associations between short-term exposure to ambient air pollution and daily hospital admissions, length of hospital stay (LOS) and hospital cost for patients with respiratory diseases and comorbid diabetes mellitus (RD-DM) in Chinese adults.

2. Exposure

Daily $PM_{2.5}$ (PM ≤2.5 μm in aerodynamic diameter), PM_{10} (PM ≤10 μm in aerodynamic diameter), sulfur dioxide (SO_2), nitrogen dioxide (NO_2), carbonic oxide (CO) and ozone (O_3) concentrations were obtained from Panzhihua Environmental Monitoring Center (http://sthjj.panzhihua.gov.cn/).

3. Outcome

Daily hospital admissions, length of hospital stay, and hospital cost for patients with RD-DM.

4. Method

The associations between air pollution and outcome (daily hospital admissions, LOS, and hospital cost) are estimated by the generalized additive model (GAM). We select log function as the link function to estimate associations of hospital admissions associated with air pollution exposure. Considering that LOS and hospital cost are continuous data and the distributions of LOS and hospital cost are approximately normal, we thus apply a GAM model with a Gaussian link for hospital cost and LOS.

5. Primary analysis

In the GAM, confounding risk factors are as follows:

- Long-term time trends: a natural smooth splined function of calendar time with 7 degrees of freedom (*df*) per year;
- Relative humidity and Temperature: a natural smooth splined function of Relative humidity and Temperature with 3 degrees of freedom (*df*) per year;
- Day of the week and public holiday: indicator variables.

Consequently, the model is as shown below:

$$logE(Y_t)\ or\ Y'_t = \alpha + \beta Z_t + ns(time, 7) + ns(TEMP, 3) + ns(HUMID, 3) + facotr(DOW_t) + factor(Holiday_t)$$

Where $E(Y_t)$ is the expected number of RD-DM patients admissions and Y'_t denotes LOS or hospital cost at day t; α is the intercept; β refers to the coefficient of air pollutants; Z_t denotes air pollution concentration at day t; *ns()* refers to a natural smooth splined function; *TEMP, HUMID* denote daily temperature and relative humidity, respectively. *DOW* and *Holiday* are an indicator of day of week and holiday effect, respectively. Considering consumer price index (*CPI*) related to hospitalization expenditure, we include *CPI* in the above model when estimating the associations between air pollution with daily hospital cost.

Primary statistics to report: we report the percentage change (PC) for admissions and absolute increase for LOS and hospital cost per 10 μg/m^3 for air pollution concentration, respectively, followed by the corresponding 95% confidence interval (95% CI). Percentage change equals relative risk minus 1 and then multiplies by 100; Absolute increase equals β (coefficient of air pollutants in the above GAM model) multiplied by 10.

Shape of association

A natural cubic spline of air pollution will be used to investigate the shape association between air

pollution and RD-DM admissions. Relative change and 95% CIs are plotted against PM.

Potential Effect modification

Effect modification by individual characteristics is investigated by stratified analyses. The subgroup variables include sex (male *vs* female), and age groups (18–64 *vs* ≥65 years).

6. Sensitivity analysis

The following sensitivity analyses will be conducted:

- Using two different pollutant models to estimate the effect values of pollutants. We included only one pollution and covariate in the single-pollution model. For the two-pollutant model, in addition to the variables in the single-pollutant model, we additionally included five additional pollutants in addition to the main pollutant;
- Changing the *df* for time (4–12 per year);
- Computing E-value at best lag day to assess the unmeasured confounding bias;
- Comparing the fixed monitoring stations data with the model-based data. we visited the China High Air Pollutants (CHAP) data, which can download model-based data for 6 air pollutants in China. We used $PM_{2.5}$ and PM_{10} data that were available for download and matched our admission data on a time scale for reanalysis.

7. Proposed Main Tables & Figures

Table 1 Summary statistics of health and environmental data during the study period.

- Average and range of annual mean hospital admissions, *LOS*, hospital cost for RD-DM
- Average and range of annual mean relative humidity
- Average and range of annual mean temperature
- Average and range of annual mean air pollution ($PM_{2.5}$, PM_{10}, SO_2, NO_2, CO, O_3) concentrations

Figure 1 The effect of six air pollutants on admission with multiple pollutant model.

Figure 2 The effect of six air pollutants on LOS with multiple pollutant model.

Figure 3 The effect of six air pollutants on hospital cost with multiple pollutant model.

Table 2 The admission attributable risk for different population with different air quality guideline.

Table 3 The LOS attributable risk for different population with different air quality guideline.

Table 4 The hospital cost attributable risk for different population with different air quality guideline.

Supplementary material: Figures

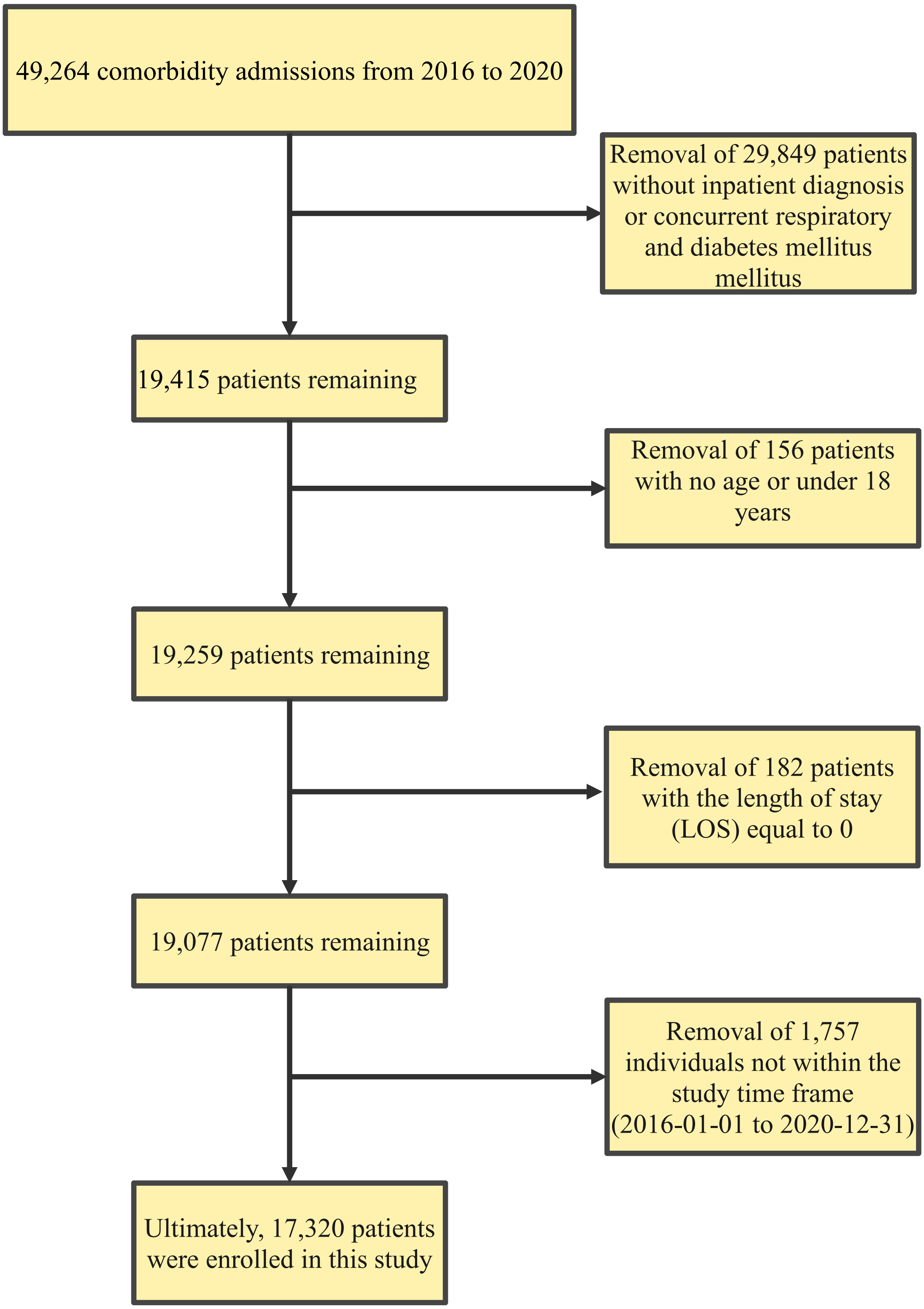

Figure S1 Flow chart of this study..

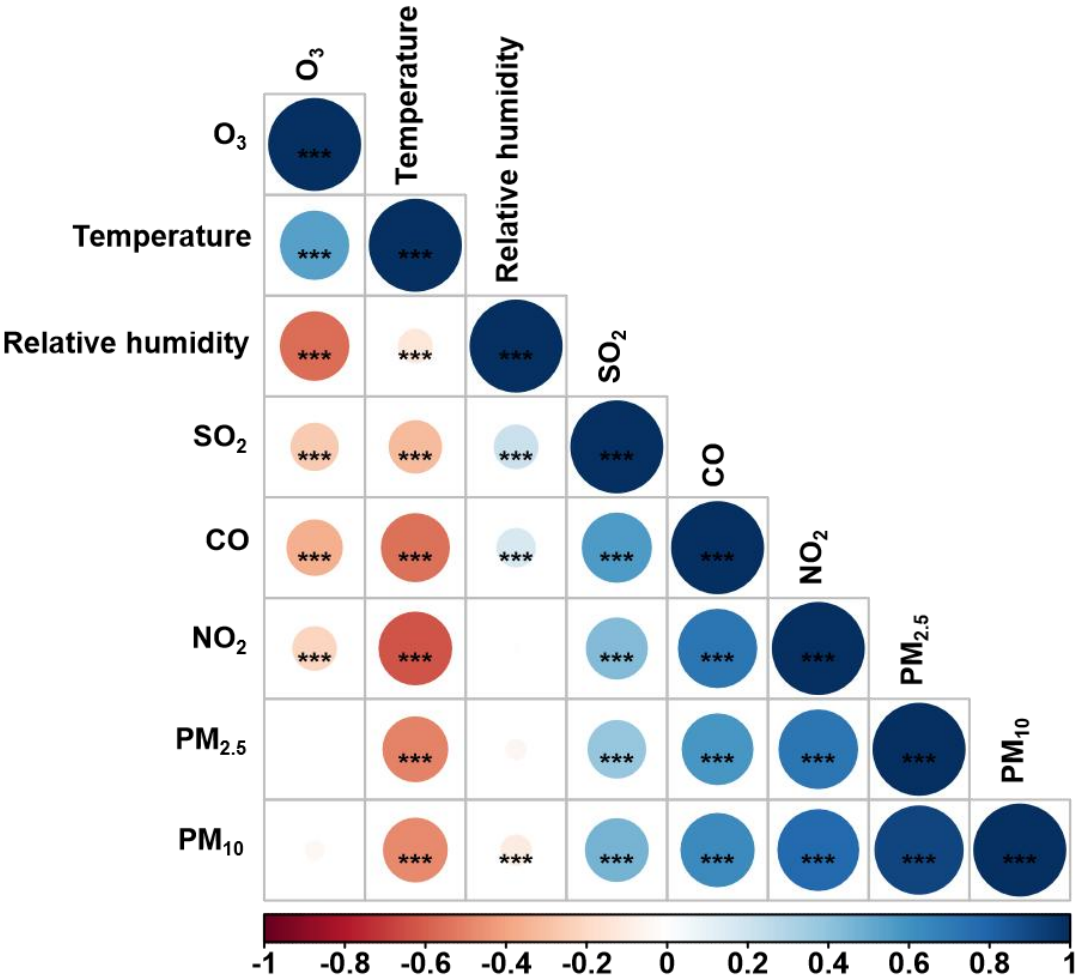

Figure S2 Spearman correlations of air pollutants and meteorological factors in Panzhihua from 2016 to 2020.

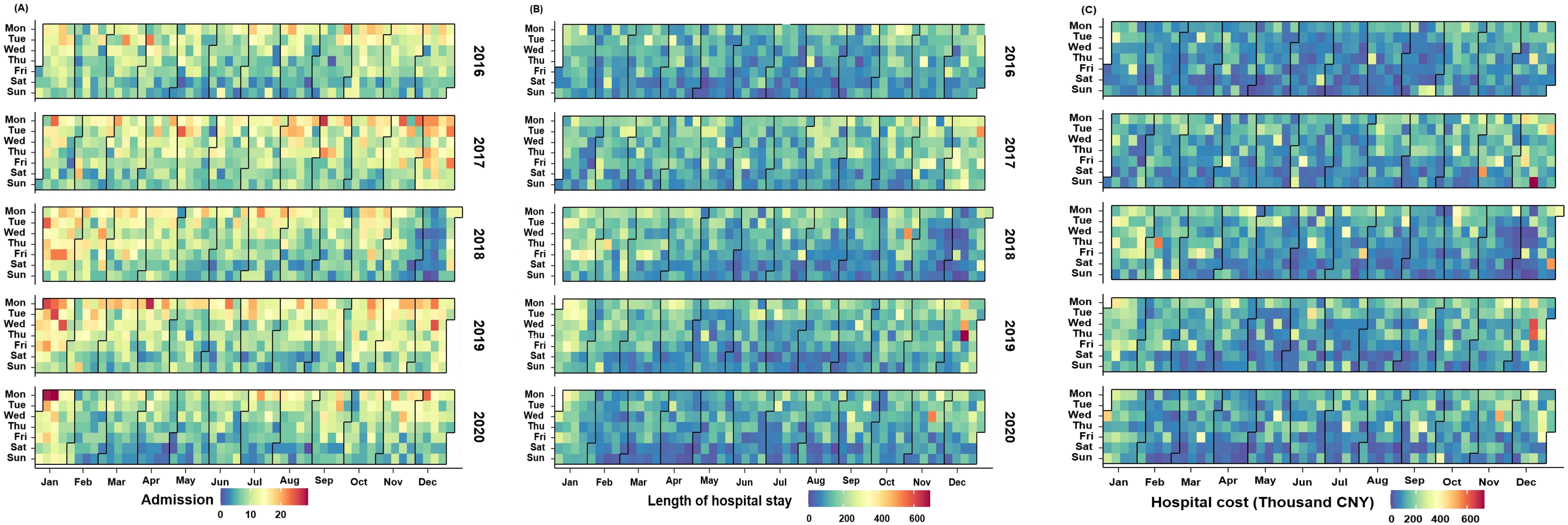

Figure S3 Calendar heat map of daily admissions, LOS, hospital cost for RD-DM in Panzhihua from January 1, 2016 to December 31, 2020.

(A): Daily RD-DM admissions;

(B): Daily RD-DM length of hospital stay;

(C): Daily RD-DM hospital cost.

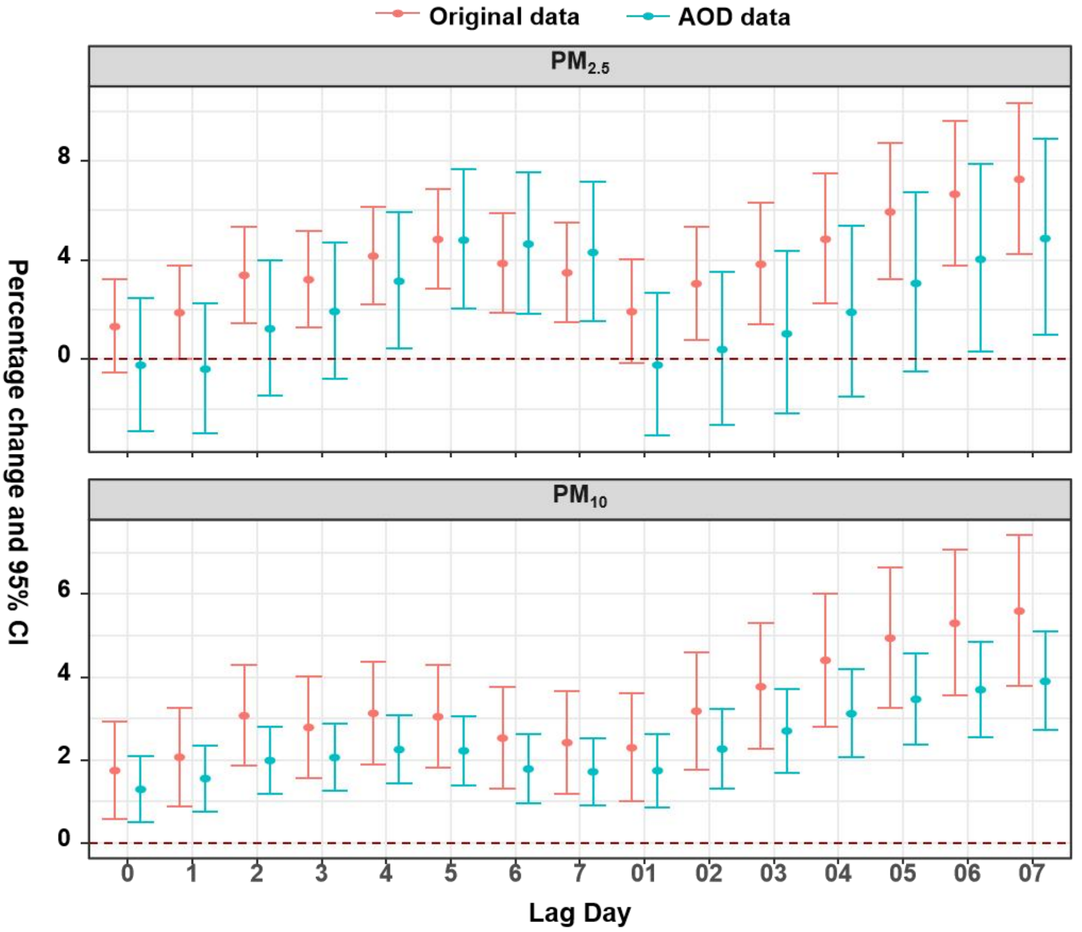

Figure S4 The effect of air pollutants on hospital admissions with single pollutant model for total population using different air pollution data.

Notes: AOD data representatives data from CHAP dataset which is only publicly available for daily average data of $PM_{2.5}$ and $PM_{10;}$ Original data indicates data from fixed monitoring stations.

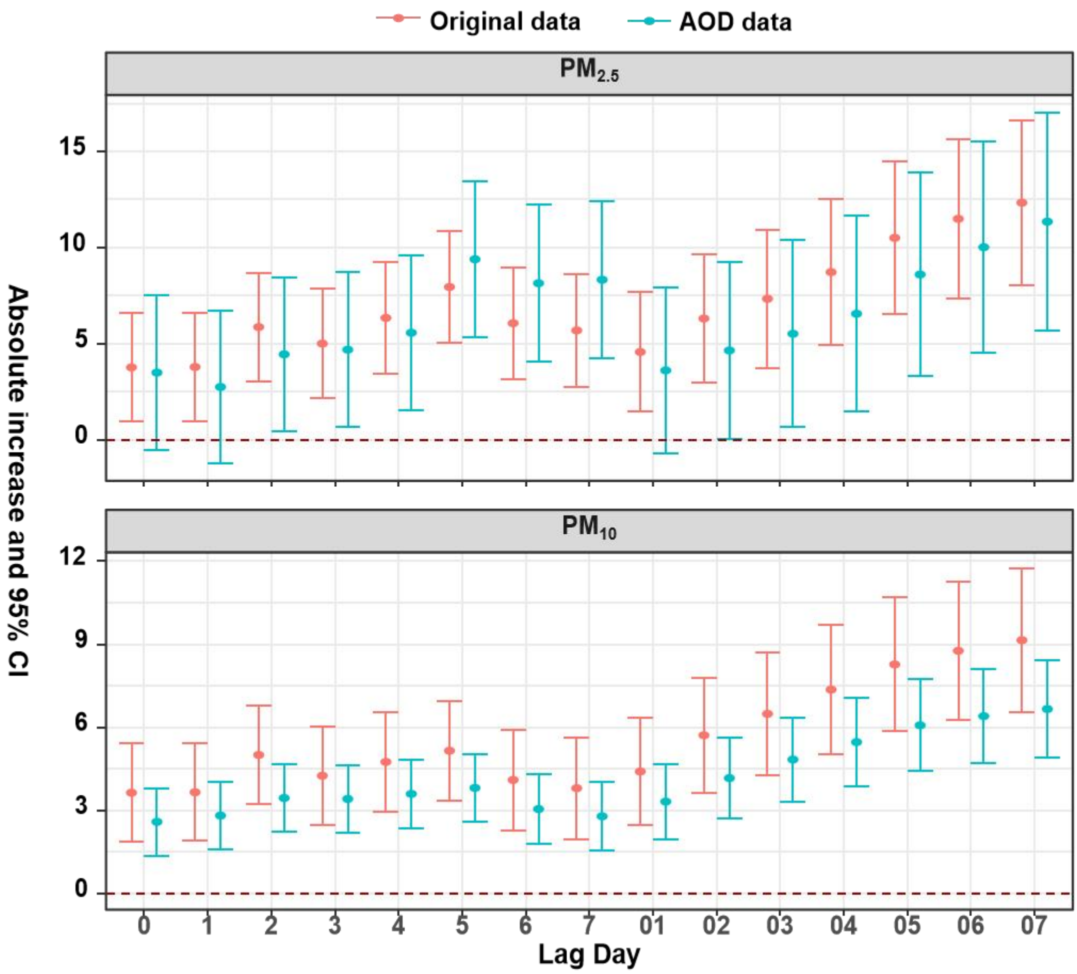

Figure S5 The effect of air pollutants on length of hospital stay (LOS) with single pollutant model for total population using different air pollution data.

Notes: AOD data representatives data from CHAP dataset which is only publicly available for daily average data of $PM_{2.5}$ and $PM_{10;}$ Original data indicates data from fixed monitoring stations.

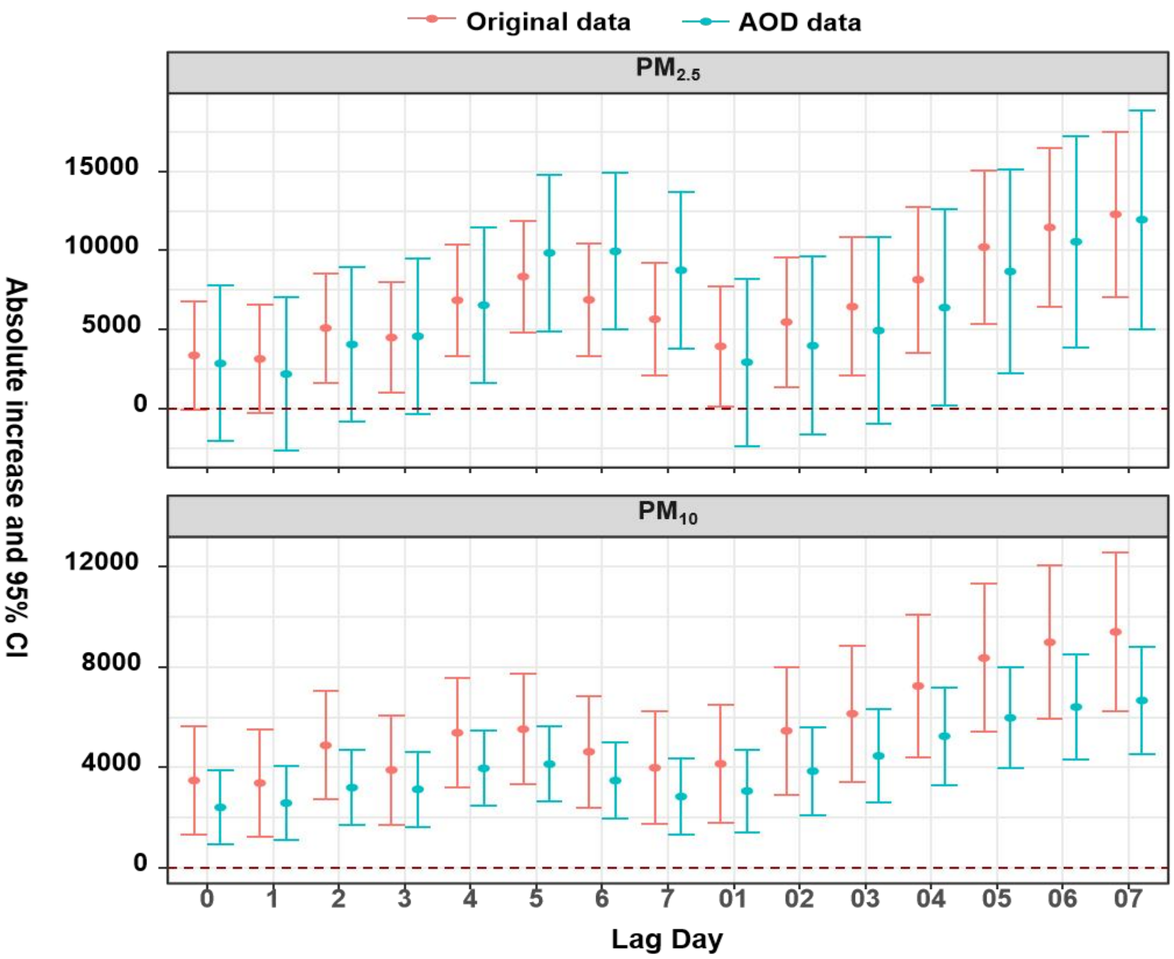

Figure S6 The effect of air pollutants on hospital cost with single pollutant model for total population using different air pollution data.

Notes: AOD data representatives data from CHAP dataset which is only publicly available for daily average data of $PM_{2.5}$ and $PM_{10;}$ Original data indicates data from fixed monitoring stations.

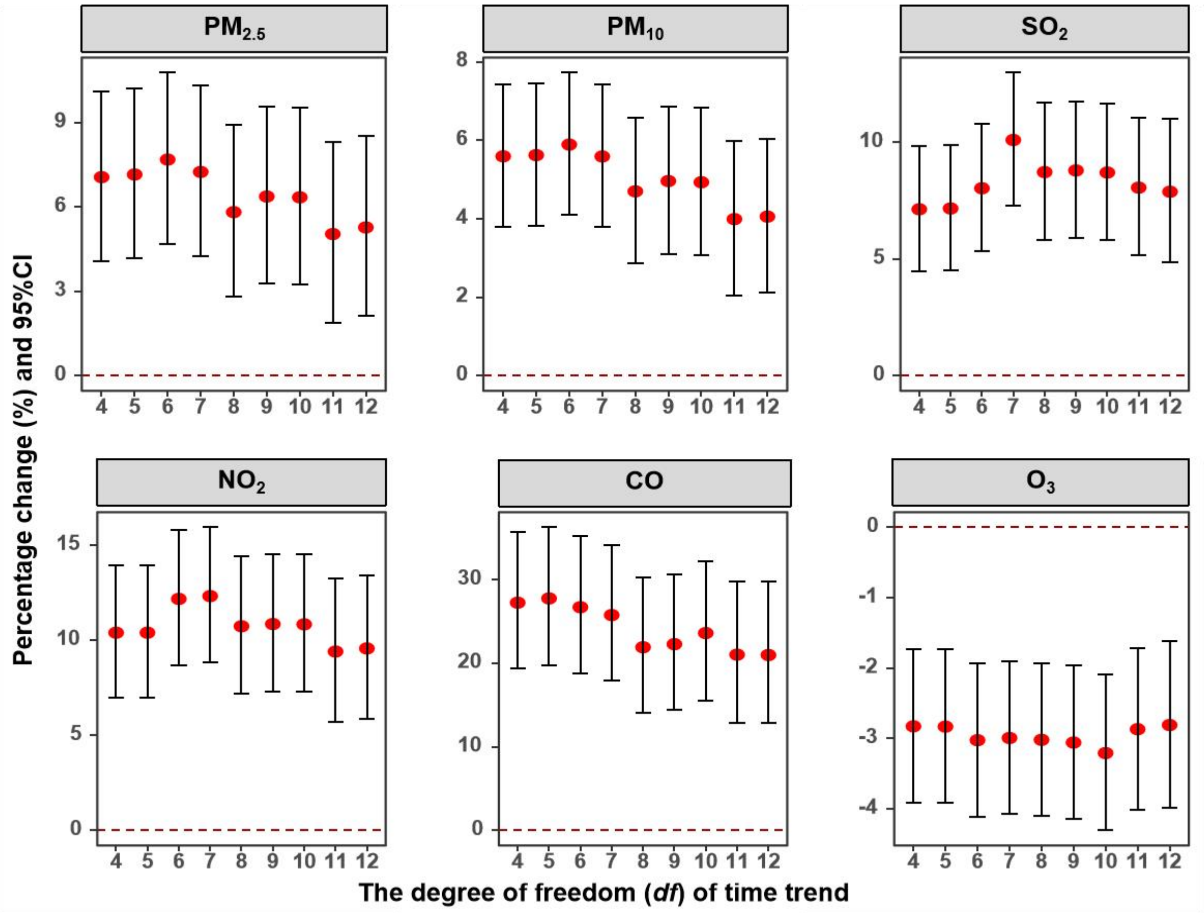

Figure S7 Percentage changes for RD-DM admissions with different degrees of freedom of time trends at lag 07 day.

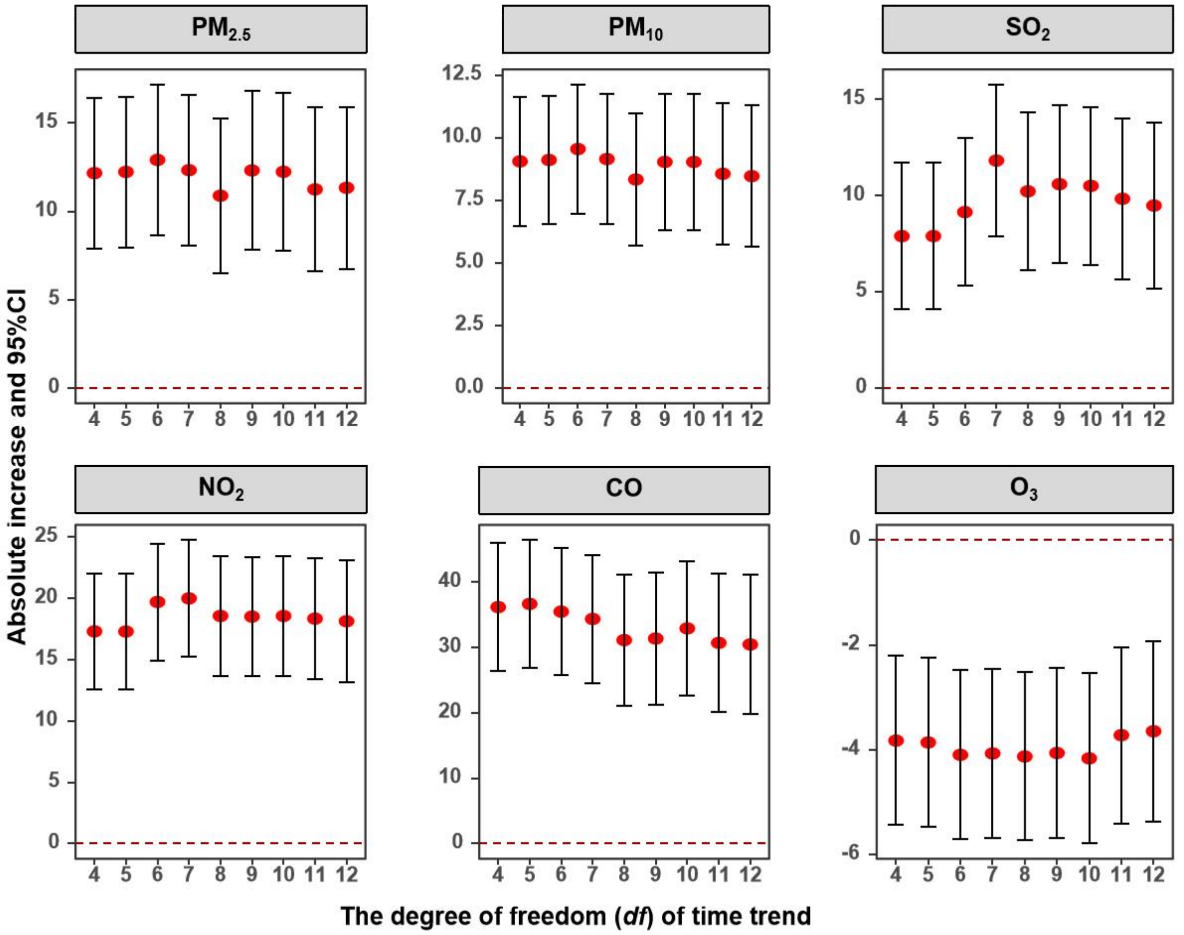

Figure S8 Absolute increase in LOS (length of hospital stay) for RD-DM with different degrees of freedom of time trends at lag 07 day.

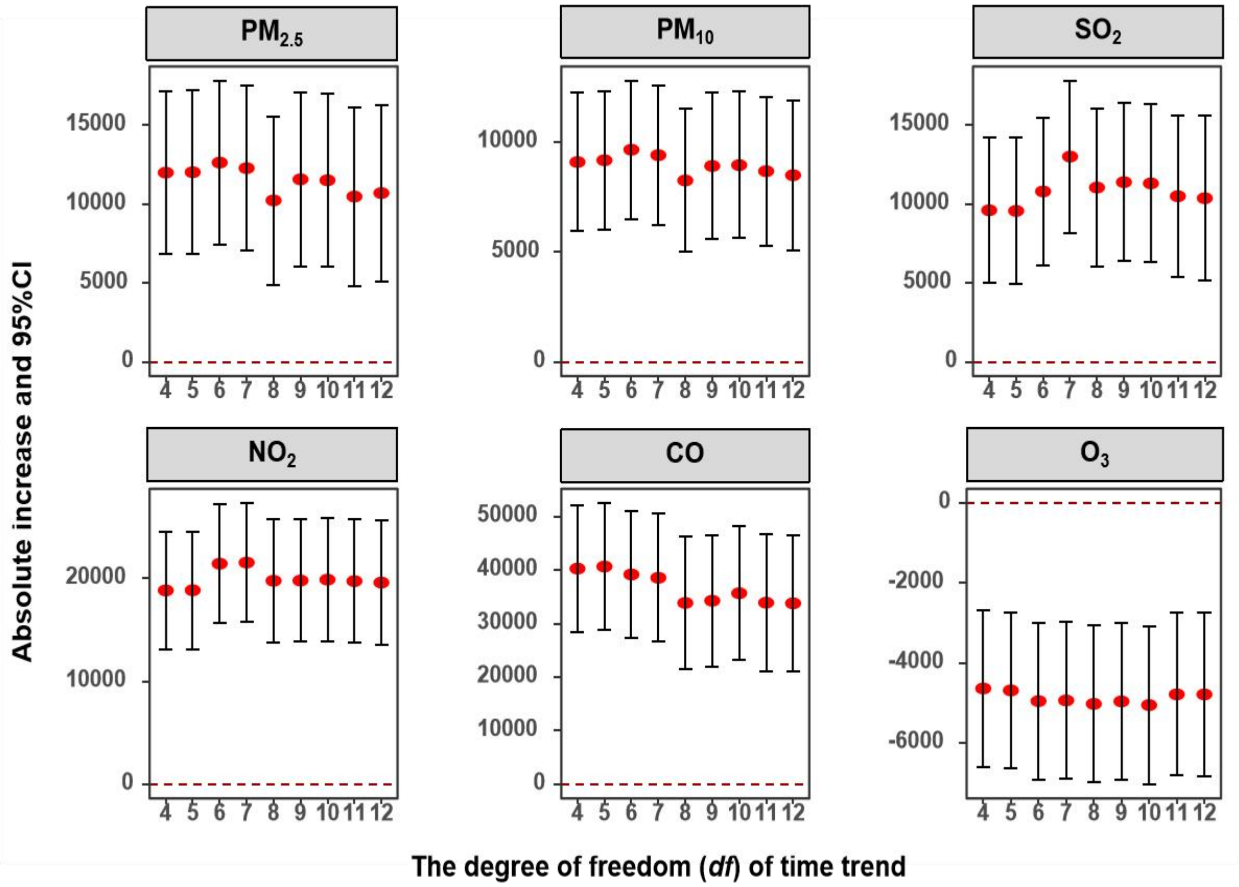

Figure S9 Absolute increase in hospital cost for RD-DM with different degrees of freedom of time trends at lag 07 day.

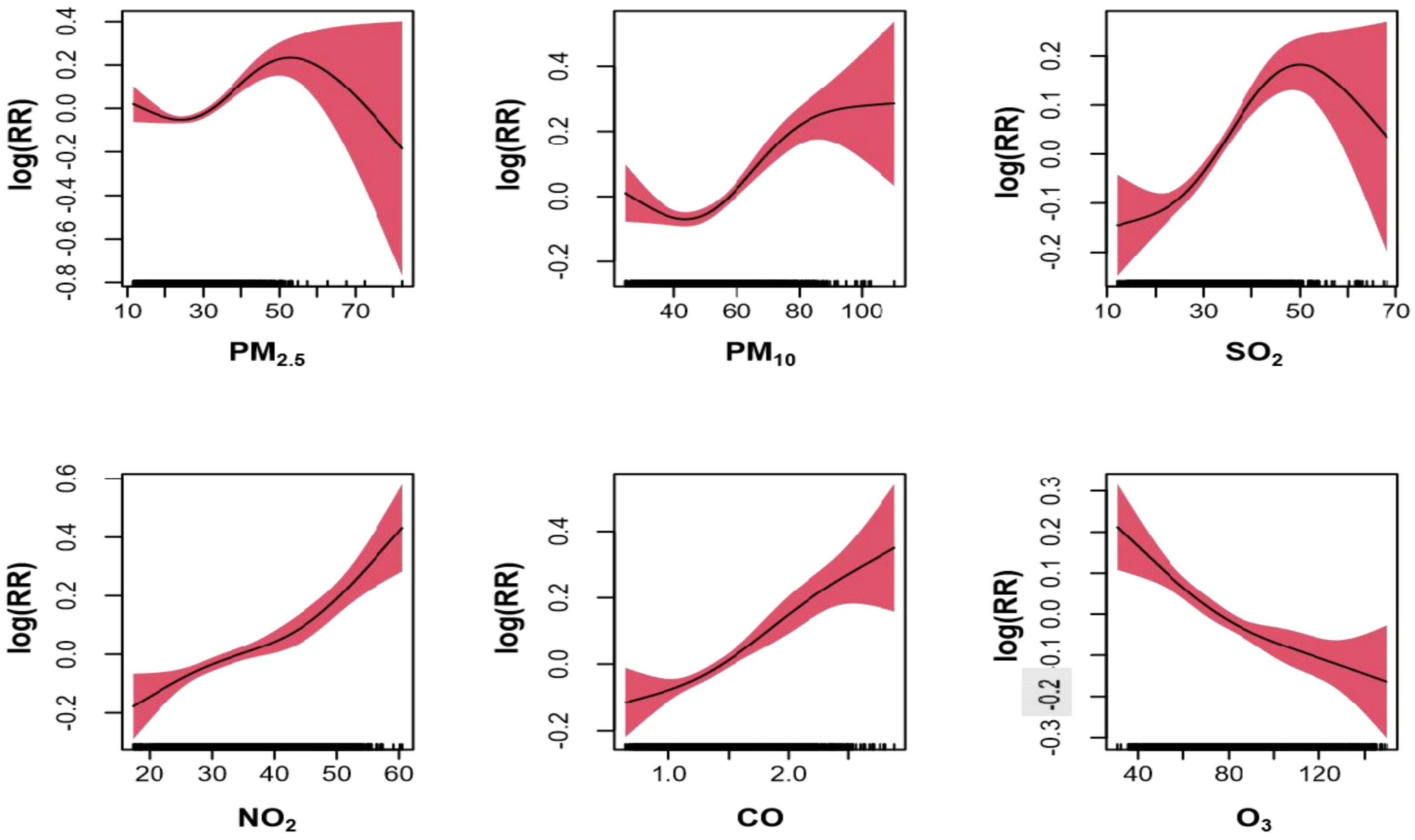

Figure S10 Dose-response relationship between RD-DM patient admissions and air pollutants.

Notes: The black rug on x-axis denotes the distribution of daily air pollutant concentration. The red shade represents a 95% confidence interval.

Supplementary material: Tables

Table S1 Demographic characteristics of different subgroups of the population

Variables	Total cases (%)	Mean	SD	Min	P25	P50	P75	Max
Total	17320.00(100.00)	9.48	5.00	0.00	6.00	9.00	12.00	29.00
Gender								
Male	10785.00 (62.27)	5.90	3.37	0.00	4.00	5.00	8.00	21.00
Female	6535.00 (37.73)	3.58	2.48	0.00	2.00	3.00	5.00	14.00
Age group								
< 65	6453.00 (37.26)	3.53	2.68	0.00	2.00	3.00	5.00	17.00
>=65	10867.00 (62.74)	5.95	3.35	0.00	4.00	5.00	8.00	22.00

Notes: SD: Standard deviation.

Table S2 The $PM_{2.5}$ effect of admissions, LOS and hospital cost for RD-DM on gender group with different lag day.

Outcome	Lag day	Male	Female	Z Value	P Value
		Increase (95% CI)	Increase (95% CI)		
Admissions	0	2.24 (0.08,4.44)	-0.15 (-2.82,2.59)	1.34	0.18
	1	3.56 (1.38,5.79)	-0.87 (-3.53,1.87)	2.48	0.01
	2	4.50 (2.26,6.78)	1.57 (-1.20,4.42)	1.58	0.11
	3	3.85 (1.60,6.15)	2.17 (-0.63,5.05)	0.90	0.37
	4	4.94 (2.66,7.27)	2.92 (0.08,5.84)	1.07	0.28
	5	4.95 (2.64,7.31)	4.71 (1.83,7.68)	0.12	0.90
	6	4.11 (1.79,6.48)	3.48 (0.58,6.45)	0.33	0.74
	7	4.13 (1.83,6.48)	2.48 (-0.37,5.42)	0.87	0.39
	01	3.50 (1.11,5.95)	-0.64 (-3.57,2.38)	2.11	0.03
	02	4.82 (2.20,7.51)	0.17 (-3.03,3.48)	2.16	0.03
	03	5.63 (2.81,8.53)	0.93 (-2.51,4.50)	2.03	0.04
	04	6.75 (3.74,9.85)	1.80 (-1.87,5.60)	2.00	0.05
	05	7.76 (4.57,11.04)	3.09 (-0.79,7.12)	1.78	0.07
	06	8.44 (5.10,11.89)	3.88 (-0.19,8.12)	1.66	0.10
	07	9.16 (5.66,12.78)	4.31 (0.05,8.74)	1.69	0.09
LOS	0	3.49 (1.20,5.78)	0.28 (-1.03,1.59)	2.38	0.02
	1	3.69 (1.40,5.98)	0.12 (-1.19,1.43)	2.65	0.01
	2	4.79 (2.48,7.09)	1.11 (-0.21,2.43)	2.71	0.01
	3	3.82 (1.48,6.16)	1.21 (-0.12,2.54)	1.90	0.06
	4	5.14 (2.79,7.50)	1.22 (-0.12,2.56)	2.84	0.00
	5	5.68 (3.32,8.05)	2.28 (0.93,3.63)	2.45	0.01
	6	3.99 (1.61,6.37)	2.08 (0.73,3.44)	1.37	0.17
	7	4.32 (1.92,6.71)	1.37 (0.00,2.73)	2.1	0.04
	01	4.35 (1.83,6.88)	0.23 (-1.21,1.67)	2.78	0.01
	02	5.64 (2.91,8.37)	0.69 (-0.87,2.25)	3.09	0.00
	03	6.32 (3.4,9.24)	1.04 (-0.63,2.71)	3.07	0.00
	04	7.43 (4.34,10.52)	1.32 (-0.45,3.08)	3.37	0.00
	05	8.64 (5.41,11.87)	1.89 (0.04,3.74)	3.56	0.00
	06	9.16 (5.80,12.53)	2.35 (0.43,4.27)	3.45	0.00
	07	9.80 (6.32,13.29)	2.54 (0.55,4.54)	3.54	0.00
Hospital cost	0	3150.88 (195.96,6105.80)	171.94 (-1329.82,1673.71)	1.76	0.08
	1	3656.96 (699.25,6614.68)	-513.66 (-2016.64,989.32)	2.46	0.01
	2	4653.19 (1675.08,7631.31)	435.09 (-1080.81,1950.99)	2.47	0.01
	3	3732.06 (719.05,6745.07)	753.52 (-778.24,2285.28)	1.73	0.08
	4	6176.13 (3141.44,9210.83)	613.45 (-933.03,2159.93)	3.20	0.00
	5	6200.18 (3157.76,9242.60)	2113.60 (559.18,3668.03)	2.34	0.02
	6	5292.10 (2234.15,8350.04)	1574.60 (11.56,3137.65)	2.12	0.03
	7	5010.92 (1933.91,8087.94)	616.91 (-957.29,2191.12)	2.49	0.01
	01	4127.66 (873.16,7382.17)	-218.63 (-1872.81,1435.54)	2.33	0.02
	02	5408.08 (1885.37,8930.79)	22.18 (-1770.07,1814.43)	2.67	0.01
	03	6090.27 (2323.50,9857.05)	298.17 (-1618.68,2215.01)	2.69	0.01
	04	7596.76 (3616.92,11576.60)	464.50 (-1563.09,2492.09)	3.13	0.00
	05	9069.47 (4905.68,13233.26)	1061.27 (-1062.67,3185.21)	3.36	0.00
	06	9943.67 (5613.08,14274.27)	1430.10 (-780.19,3640.39)	3.43	0.00
	07	10715.20 (6221.27,15209.13)	1484.73 (-810.32,3779.78)	3.59	0.00

Table S3 The $PM_{2.5}$ effect of admissions, LOS and hospital cost for RD-DM on age group with different lag day.

Outcome	Lag day	Younger adult Increase (95% CI)	The elderly Increase (95% CI)	Z Value	P Value
Admissions	0	-0.56(-3.33,2.29)	2.65(0.49,4.85)	1.76	0.08
	1	-0.54(-3.33,2.34)	3.46(1.29,5.69)	2.18	0.03
	2	-0.43(-3.30,2.53)	5.72(3.48,8.00)	3.24	0.00
	3	-0.14(-3.03,2.84)	5.19(2.94,7.48)	2.79	0.01
	4	1.52(-1.43,4.56)	5.71(3.44,8.03)	2.17	0.03
	5	2.84(-0.14,5.92)	6.01(3.71,8.37)	1.62	0.11
	6	4.01(0.97,7.14)	3.82(1.52,6.18)	0.09	0.92
	7	3.48(0.49,6.56)	3.61(1.31,5.95)	0.06	0.95
	01	-0.69(-3.73,2.46)	3.70(1.30,6.15)	2.17	0.03
	02	-0.76(-4.08,2.67)	5.54(2.92,8.24)	2.86	0.00
	03	-0.73(-4.27,2.94)	6.77(3.94,9.68)	3.16	0.00
	04	-0.18(-3.93,3.72)	8.05(5.03,11.17)	3.26	0.00
	05	0.71(-3.25,4.82)	9.29(6.09,12.59)	3.21	0.00
	06	1.86(-2.30,6.20)	9.75(6.39,13.22)	2.81	0.00
	07	2.66(-1.69,7.20)	10.26(6.74,13.89)	2.59	0.01
LOS	0	0.33(-0.79,1.44)	3.52(1.03,6.01)	2.29	0.02
	1	0.70(-0.42,1.82)	3.17(0.68,5.66)	1.77	0.08
	2	0.38(-0.74,1.51)	5.55(3.05,8.06)	3.69	0.00
	3	0.53(-0.61,1.67)	4.54(2.01,7.08)	2.83	0.00
	4	1.02(-0.14,2.17)	5.40(2.85,7.95)	3.07	0.00
	5	1.28(0.12,2.44)	6.73(4.17,9.30)	3.80	0.00
	6	1.81(0.65,2.97)	4.30(1.71,6.88)	1.72	0.09
	7	1.54(0.37,2.71)	4.20(1.59,6.8)	1.83	0.07
	01	0.61(-0.61,1.84)	4.04(1.30,6.78)	2.24	0.03
	02	0.65(-0.68,1.98)	5.75(2.78,8.72)	3.07	0.00
	03	0.76(-0.67,2.18)	6.68(3.51,9.86)	3.34	0.00
	04	1.03(-0.48,2.54)	7.81(4.46,11.17)	3.62	0.00
	05	1.36(-0.22,2.93)	9.28(5.77,12.78)	4.04	0.00
	06	1.81(0.17,3.45)	9.81(6.16,13.46)	3.92	0.00
	07	2.11(0.40,3.81)	10.35(6.56,14.14)	3.89	0.00
Hospital cost	0	381.71(-984.89,1748.31)	2943.45(-185.16,6072.06)	1.47	0.14
	1	541.14(-828.33,1910.61)	2604.22(-527.63,5736.08)	1.18	0.24
	2	-221.88(-1604.07,1160.31)	5349.82(2201.75,8497.89)	3.18	0.00
	3	380.21(-1017.70,1778.11)	4130.82(945.37,7316.28)	2.11	0.03
	4	1113.23(-297.99,2524.46)	5696.28(2484.64,8907.91)	2.56	0.01
	5	1589.48(170.23,3008.73)	6736.96(3513.78,9960.13)	2.86	0.00
	6	2674.20(1251.80,4096.61)	4187.91(943.50,7432.33)	0.84	0.40
	7	1453.38(17.89,2888.87)	4184.46(921.02,7447.90)	1.50	0.13
	01	554.51(-949.92,2058.95)	3351.64(-96.18,6799.45)	1.46	0.15
	02	324.73(-1304.61,1954.06)	5128.03(1396.59,8859.47)	2.31	0.02
	03	420.17(-1321.83,2162.16)	6004.09(2014.34,9993.85)	2.51	0.01
	04	757.77(-1084.25,2599.80)	7347.23(3130.25,11564.22)	2.81	0.01
	05	1222.45(-706.85,3151.75)	8959.03(4547.15,13370.9)	3.15	0.00
	06	1914.30(-92.48,3921.09)	9494.38(4903.23,14085.53)	2.97	0.00
	07	2183.96(100.82,4267.10)	10039.57(5273.49,14805.66)	2.96	0.00

Table S4 The PM_{10} effect of admissions, LOS and hospital cost for RD-DM on gender group with different lag day.

Outcome	Lag day	Male Increase (95% CI)	Female Increase (95% CI)	Z Value	P Value
Admissions	0	2.59(1.23,3.97)	0.44(-1.25,2.16)	1.93	0.05
	1	3.47(2.10,4.86)	-0.20(-1.89,1.52)	3.27	0.00
	2	3.92(2.52,5.33)	1.70(-0.04,3.48)	1.93	0.05
	3	3.40(2.00,4.83)	1.79(0.04,3.58)	1.39	0.16
	4	3.66(2.23,5.10)	2.32(0.54,4.14)	1.14	0.26
	5	3.14(1.71,4.59)	2.97(1.18,4.79)	0.14	0.89
	6	2.90(1.47,4.35)	2.00(0.22,3.81)	0.77	0.44
	7	3.00(1.58,4.44)	1.58(-0.18,3.38)	1.21	0.23
	01	3.66(2.16,5.19)	0.12(-1.73,2.01)	2.87	0.00
	02	4.64(3.01,6.30)	0.85(-1.17,2.91)	2.82	0.00
	03	5.26(3.51,7.04)	1.40(-0.76,3.61)	2.68	0.01
	04	5.89(4.04,7.78)	2.02(-0.26,4.36)	2.54	0.01
	05	6.32(4.38,8.30)	2.73(0.34,5.18)	2.25	0.02
	06	6.69(4.67,8.75)	3.11(0.61,5.67)	2.15	0.03
	07	7.07(4.97,9.22)	3.30(0.72,5.95)	2.19	0.03
LOS	0	3.17(1.73,4.62)	0.46(-0.36,1.29)	3.20	0.00
	1	3.46(2.03,4.89)	0.20(-0.62,1.02)	3.87	0.00
	2	3.94(2.50,5.38)	1.07(0.24,1.89)	3.39	0.00
	3	3.31(1.85,4.77)	0.94(0.11,1.78)	2.76	0.01
	4	3.75(2.28,5.22)	1.00(0.16,1.83)	3.19	0.00
	5	3.64(2.16,5.12)	1.51(0.67,2.35)	2.45	0.01
	6	2.78(1.29,4.27)	1.31(0.46,2.16)	1.68	0.09
	7	2.96(1.46,4.46)	0.83(-0.03,1.68)	2.42	0.02
	01	4.02(2.44,5.60)	0.39(-0.51,1.29)	3.90	0.00
	02	4.93(3.23,6.62)	0.79(-0.18,1.76)	4.15	0.00
	03	5.46(3.66,7.27)	1.03(-0.01,2.06)	4.18	0.00
	04	6.10(4.2,7.99)	1.26(0.17,2.35)	4.33	0.00
	05	6.68(4.70,8.65)	1.59(0.46,2.72)	4.38	0.00
	06	6.92(4.87,8.97)	1.84(0.66,3.01)	4.22	0.00
	07	7.22(5.11,9.34)	1.91(0.70,3.13)	4.27	0.00
Hospital cost	0	3218.44(1358.38,5078.50)	207.44(-739.23,1154.11)	2.83	0.00
	1	3588.71(1740.52,5436.90)	-237.90(-1179.62,703.81)	3.62	0.00
	2	4262.98(2404.09,6121.87)	597.18(-351.96,1546.32)	3.44	0.00
	3	3286.27(1402.27,5170.27)	585.83(-374.07,1545.74)	2.50	0.01
	4	4647.11(2754.17,6540.06)	685.79(-281.37,1652.95)	3.65	0.00
	5	4187.75(2287.17,6088.33)	1287.90(314.59,2261.20)	2.66	0.01
	6	3741.65(1829.11,5654.18)	847.78(-132.59,1828.16)	2.64	0.01
	7	3436.87(1515.71,5358.03)	504.99(-480.58,1490.57)	2.66	0.01
	01	4131.01(2094.44,6167.59)	-28.65(-1067.23,1009.92)	3.57	0.00
	02	5174.60(2987.20,7362.01)	247.57(-870.37,1365.51)	3.93	0.00
	03	5665.36(3337.97,7992.75)	430.17(-759.84,1620.18)	3.93	0.00
	04	6597.06 (4154.75,9039.37)	613.83 (-637.80,1865.45)	4.27	0.00
	05	7387.25(4846.13,9928.37)	929.32(-374.87,2233.52)	4.43	0.00
	06	7846.70(5213.51,10479.88)	1087.80(-264.49,2440.10)	4.48	0.00
	07	8208.18(5488.33,10928.04)	1127.56(-270.05,2525.16)	4.54	0.00

Table S5 The PM_{10} effect of admissions, LOS and hospital cost for RD-DM on age group with different lag day.

Outcome	Lag day	Younger adult Increase (95% CI)	The elderly Increase (95% CI)	Z Value	P Value
Admissions	0	0.07(-1.70,1.87)	2.87(1.52,4.24)	2.44	0.01
	1	-0.21(-1.99,1.60)	3.46(2.11,4.83)	3.17	0.00
	2	0.16(-1.67,2.01)	4.76(3.38,6.16)	3.89	0.00
	3	0.17(-1.66,2.04)	4.27(2.88,5.67)	3.44	0.00
	4	1.16(-0.72,3.06)	4.24(2.83,5.67)	2.55	0.01
	5	1.72(-0.15,3.63)	3.83(2.40,5.27)	1.73	0.08
	6	2.47(0.60,4.38)	2.63(1.21,4.06)	0.13	0.9
	7	2.21(0.35,4.10)	2.65(1.24,4.07)	0.36	0.72
	01	-0.10(-2.05,1.88)	3.83(2.33,5.34)	3.09	0.00
	02	-0.03(-2.14,2.13)	5.16(3.53,6.80)	3.76	0.00
	03	0.03(-2.21,2.32)	6.03(4.29,7.79)	4.07	0.00
	04	0.42(-1.94,2.84)	6.77(4.93,8.64)	4.08	0.00
	05	0.91(-1.56,3.44)	7.3(5.38,9.26)	3.92	0.00
	06	1.60(-0.98,4.25)	7.47(5.46,9.52)	3.45	0.00
	07	2.06(-0.62,4.81)	7.69(5.61,9.81)	3.19	0.00
LOS	0	0.37(-0.34,1.07)	3.34(1.77,4.90)	3.39	0.00
	1	0.42(-0.28,1.12)	3.28(1.73,4.84)	3.29	0.00
	2	0.32(-0.38,1.03)	4.72(3.16,6.28)	5.03	0.00
	3	0.32(-0.40,1.03)	3.98(2.39,5.56)	4.13	0.00
	4	0.60(-0.12,1.32)	4.20(2.61,5.79)	4.03	0.00
	5	0.70(-0.03,1.42)	4.52(2.91,6.12)	4.26	0.00
	6	1.03(0.30,1.75)	3.12(1.50,4.74)	2.31	0.02
	7	0.81(0.07,1.54)	3.05(1.43,4.68)	2.47	0.01
	01	0.47(-0.3,1.25)	4.00(2.28,5.72)	3.67	0.00
	02	0.51(-0.32,1.34)	5.28(3.43,7.12)	4.62	0.00
	03	0.55(-0.33,1.43)	6.01(4.05,7.97)	4.98	0.00
	04	0.69(-0.24,1.62)	6.76(4.70,8.82)	5.27	0.00
	05	0.84(-0.12,1.81)	7.53(5.38,9.67)	5.57	0.00
	06	1.08(0.08,2.08)	7.79(5.57,10.01)	5.39	0.00
	07	1.21(0.17,2.24)	8.05(5.76,10.35)	5.33	0.00
Hospital	0	438.28(-422.24,1298.8)	2966.91(994.08,4939.75)	2.30	0.02
	1	319.47(-537.54,1176.48)	3022.90(1063.81,4982.00)	2.48	0.01
	2	-109.23(-973.90,755.44)	4981.34(3017.07,6945.61)	4.65	0.00
	3	208.55(-666.38,1083.47)	3661.68(1669.09,5654.27)	3.11	0.00
	4	717.38(-164.05,1598.81)	4603.46(2598.58,6608.33)	3.48	0.00
	5	694.14(-193.56,1581.85)	4780.85(2765.59,6796.11)	3.64	0.00
	6	1376.34(484.73,2267.95)	3212.06(1179.58,5244.54)	1.62	0.10
	7	617.86(-280.00,1515.71)	3342.97(1302.17,5383.77)	2.40	0.02
	01	454.47(-488.80,1397.75)	3625.75(1463.82,5787.67)	2.64	0.01
	02	296.78(-718.10,1311.66)	5126.52(2806.74,7446.30)	3.74	0.00
	03	329.68(-750.10,1409.45)	5771.07(3303.18,8238.95)	3.96	0.00
	04	525.49(-609.70,1660.67)	6684.88(4093.21,9276.55)	4.27	0.00
	05	713.97(-468.76,1896.69)	7605.57(4908.93,10302.22)	4.59	0.00
	06	1045.84(-179.96,2271.63)	7874.81(5076.97,10672.65)	4.38	0.00
	07	1129.38(-137.20,2395.96)	8189.77(5297.82,11081.73)	4.38	0.00

Table S6 The SO_2 effect of admissions, LOS and hospital cost for RD-DM on gender group with different lag day.

Outcome	Lag day	Male	Female	Z Value	P Value
		Increase (95% CI)	Increase (95% CI)		
Admissions	0	4.79(2.99,6.62)	2.58(0.32,4.90)	1.47	0.14
	1	3.30(1.51,5.13)	1.02(-1.22,3.32)	1.54	0.12
	2	2.98(1.23,4.76)	2.45(0.26,4.68)	0.37	0.71
	3	3.93(2.17,5.71)	3.62(1.42,5.87)	0.21	0.83
	4	5.46(3.69,7.27)	3.91(1.67,6.19)	1.05	0.29
	5	3.97(2.18,5.79)	3.36(1.12,5.66)	0.41	0.68
	6	2.46(0.71,4.24)	1.81(-0.38,4.04)	0.46	0.65
	7	2.77(0.99,4.57)	1.14(-1.07,3.41)	1.11	0.27
	01	5.86(3.67,8.10)	2.60(-0.11,5.39)	1.80	0.07
	02	6.72(4.27,9.24)	3.72(0.68,6.84)	1.49	0.14
	03	8.10(5.41,10.85)	5.29(1.97,8.71)	1.27	0.21
	04	10.06(7.17,13.04)	6.77(3.19,10.46)	1.38	0.17
	05	11.07(8.00,14.22)	7.79(4.00,11.71)	1.29	0.20
	06	11.16(8.00,14.42)	7.87(3.96,11.91)	1.26	0.21
	07	11.43(8.16,14.79)	7.7(3.70,11.87)	1.38	0.17
LOS	0	4.62(2.72,6.52)	0.95(-0.14,2.03)	3.29	0.00
	1	2.65(0.78,4.51)	0.68(-0.39,1.74)	1.80	0.07
	2	2.67(0.81,4.54)	1.47(0.41,2.53)	1.10	0.27
	3	2.91(1.04,4.78)	1.64(0.58,2.70)	1.16	0.25
	4	4.42(2.55,6.29)	1.50(0.44,2.56)	2.66	0.01
	5	2.76(0.88,4.64)	0.85(-0.22,1.92)	1.73	0.08
	6	0.87(-1.01,2.75)	0.9(-0.17,1.97)	0.02	0.98
	7	1.44(-0.43,3.31)	0.85(-0.21,1.91)	0.54	0.59
	01	5.23(2.97,7.49)	1.17(-0.12,2.46)	3.05	0.00
	02	5.99(3.47,8.51)	1.88(0.45,3.32)	2.77	0.01
	03	6.82(4.09,9.54)	2.54(0.98,4.09)	2.68	0.01
	04	8.24(5.37,11.12)	2.99(1.35,4.63)	3.11	0.00
	05	8.76(5.74,11.77)	3.11(1.39,4.83)	3.19	0.00
	06	8.42(5.29,11.56)	3.23(1.45,5.02)	2.82	0.00
	07	8.36(5.14,11.59)	3.32(1.48,5.15)	2.66	0.01
Hospital cost	0	5056.91(2607.17,7506.66)	345.17(-901.84,1592.17)	3.36	0.00
	1	3160.67(758.77,5562.56)	-12.61(-1232.86,1207.63)	2.31	0.02
	2	4049.01(1650.93,6447.10)	1198.40(-20.84,2417.63)	2.08	0.04
	3	3790.33(1385.78,6194.87)	1368.71(147.21,2590.20)	1.76	0.08
	4	5149.27(2742.30,7556.23)	1148.84(-75.24,2372.91)	2.90	0.00
	5	3108.53(698.07,5519)	860.59(-369.34,2090.53)	1.63	0.10
	6	676.53(-1725.44,3078.50)	558.44(-668.47,1785.35)	0.09	0.93
	7	2064.50(-331.13,4460.14)	630.49(-592.58,1853.55)	1.04	0.30
	01	5961.75(3050.31,8873.19)	236.55(-1243.24,1716.33)	3.44	0.00
	02	7495.54(4253.42,10737.66)	939.21(-709.25,2587.66)	3.53	0.00
	03	8681.44(5178.45,12184.43)	1563.82(-217.63,3345.28)	3.55	0.00
	04	10351.82(6650.91,14052.73)	1960.01(75.11,3844.90)	3.96	0.00
	05	10889.61(7011.87,14767.35)	2175.63(201.62,4149.63)	3.93	0.00
	06	10359.14(6334.57,14383.70)	2237.32(191.66,4282.99)	3.53	0.00
	07	10412.59(6265.93,14559.26)	2317.90(212.09,4423.71)	3.41	0.00

Table S7 The SO_2 effect of admissions, LOS and hospital cost for RD-DM on age group with different lag day.

Outcome	Lag day	Younger adult Increase (95% CI)	The elderly Increase (95% CI)	Z Value	P Value
Admissions	0	2.37(-0.02,4.81)	4.92(3.14,6.73)	1.66	0.10
	1	1.43(-0.92,3.84)	3.13(1.35,4.93)	1.11	0.27
	2	1.22(-1.05,3.55)	3.74(2.00,5.51)	1.70	0.09
	3	2.91(0.61,5.26)	4.30(2.56,6.07)	0.93	0.35
	4	3.80(1.47,6.18)	5.45(3.69,7.24)	1.10	0.27
	5	3.89(1.55,6.28)	3.60(1.82,5.41)	0.19	0.85
	6	1.71(-0.57,4.04)	2.59(0.85,4.37)	0.60	0.55
	7	1.48(-0.84,3.85)	2.62(0.86,4.41)	0.76	0.45
	01	2.72(-0.13,5.64)	5.83(3.66,8.05)	1.68	0.09
	02	2.97(-0.17,6.21)	7.18(4.74,9.68)	2.04	0.04
	03	4.15(0.75,7.67)	8.70(6.03,11.44)	2.02	0.04
	04	5.56(1.90,9.34)	10.58(7.71,13.53)	2.07	0.04
	05	6.78(2.92,10.79)	11.35(8.32,14.48)	1.78	0.07
	06	6.81(2.84,10.93)	11.49(8.35,14.73)	1.77	0.08
	07	6.78(2.71,11.01)	11.68(8.43,15.02)	1.80	0.07
LOS	0	0.86(-0.07,1.78)	4.76(2.70,6.83)	3.38	0.00
	1	0.60(-0.30,1.50)	2.76(0.74,4.79)	1.91	0.06
	2	0.25(-0.65,1.15)	3.94(1.92,5.97)	3.27	0.00
	3	0.85(-0.05,1.75)	3.76(1.73,5.78)	2.57	0.01
	4	1.26(0.36,2.17)	4.71(2.69,6.74)	3.05	0.00
	5	1.19(0.28,2.10)	2.47(0.43,4.52)	1.13	0.26
	6	0.37(-0.53,1.28)	1.48(-0.56,3.52)	0.97	0.33
	7	0.52(-0.38,1.42)	1.85(-0.18,3.89)	1.18	0.24
	01	1.04(-0.05,2.14)	5.42(2.96,7.88)	3.19	0.00
	02	1.01(-0.20,2.22)	6.93(4.19,9.66)	3.88	0.00
	03	1.32(0.02,2.63)	8.08(5.13,11.04)	4.10	0.00
	04	1.77(0.40,3.15)	9.50(6.38,12.63)	4.44	0.00
	05	2.11(0.67,3.55)	9.77(6.49,13.04)	4.20	0.00
	06	2.07(0.58,3.55)	9.60(6.20,12.99)	3.98	0.00
	07	2.08(0.56,3.61)	9.60(6.10,13.10)	3.86	0.00
Hospital cost	0	1180.96(52.84,2309.08)	4333.93(1735.61,6932.24)	2.18	0.03
	1	630.12(-475.23,1735.47)	2642.32(97.76,5186.87)	1.42	0.16
	2	-83.06(-1187.52,1021.40)	5480.04(2947.38,8012.69)	3.95	0.00
	3	1061.60(-43.98,2167.18)	4198.95(1655.19,6742.71)	2.22	0.03
	4	1526.90(420.45,2633.36)	4835.01(2286.47,7383.54)	2.33	0.02
	5	1074.65(-36.80,2186.10)	3012.87(453.45,5572.29)	1.36	0.17
	6	825.73(-283.00,1934.45)	577.73(-1976.16,3131.63)	0.17	0.86
	7	389.25(-716.12,1494.62)	2472.63(-71.43,5016.69)	1.47	0.14
	01	1285.08(-47.87,2618.02)	5056.90(1968.15,8145.64)	2.20	0.03
	02	1003.31(-475.27,2481.89)	7636.41(4203.12,11069.70)	3.48	0.00
	03	1417.95(-172.32,3008.22)	9018.65(5311.31,12725.99)	3.69	0.00
	04	1962.79(287.48,3638.10)	10476.80(6558.65,14394.96)	3.92	0.00
	05	2220.63(472.45,3968.81)	10943.71(6838.11,15049.31)	3.83	0.00
	06	2336.74(530.57,4142.90)	10344.68(6083.52,14605.85)	3.39	0.00
	07	2280.84(426.45,4135.22)	10520.59(6129.73,14911.45)	3.39	0.00

Table S8 The NO_2 effect of admissions, LOS and hospital cost for RD-DM on gender group with different lag day.

Outcome	Lag day	Male Increase (95% CI)	Female Increase (95% CI)	Z Value	P Value
Admissions	0	6.14(3.26,9.10)	2.68(-0.85,6.33)	1.46	0.14
	1	7.73(4.91,10.64)	1.69(-1.70,5.19)	2.63	0.01
	2	8.79(5.94,11.72)	3.93(0.44,7.53)	2.07	0.04
	3	8.51(5.64,11.45)	5.04(1.49,8.71)	1.46	0.14
	4	8.04(5.12,11.03)	6.45(2.78,10.24)	0.65	0.51
	5	7.89(4.96,10.91)	7.20(3.51,11.02)	0.28	0.78
	6	6.96(4.06,9.94)	5.74(2.14,9.46)	0.51	0.61
	7	6.91(4.02,9.88)	4.29(0.76,7.95)	1.10	0.27
	01	8.10(5.00,11.29)	2.49(-1.24,6.36)	2.22	0.03
	02	9.95(6.64,13.36)	3.61(-0.38,7.75)	2.34	0.02
	03	11.31(7.81,14.93)	4.86(0.64,9.27)	2.25	0.02
	04	12.31(8.64,16.11)	6.25(1.79,10.90)	2.01	0.04
	05	13.16(9.33,17.12)	7.56(2.90,12.43)	1.78	0.08
	06	13.68(9.74,17.77)	8.37(3.55,13.41)	1.63	0.10
	07	14.17(10.12,18.37)	8.69(3.74,13.87)	1.64	0.10
LOS	0	7.59(4.59,10.58)	2.19(0.48,3.90)	3.07	0.00
	1	7.94(5.07,10.81)	1.93(0.29,3.57)	3.56	0.00
	2	8.98(6.12,11.84)	3.10(1.46,4.73)	3.50	0.00
	3	9.48(6.59,12.37)	3.00(1.34,4.66)	3.81	0.00
	4	8.59(5.67,11.51)	3.12(1.45,4.79)	3.18	0.00
	5	8.72(5.79,11.64)	3.47(1.80,5.14)	3.06	0.00
	6	6.97(4.03,9.91)	3.42(1.75,5.10)	2.06	0.04
	7	6.10(3.15,9.05)	2.37(0.68,4.05)	2.15	0.03
	01	9.05(5.89,12.21)	2.38(0.57,4.18)	3.59	0.00
	02	10.69(7.38,14.01)	3.13(1.23,5.03)	3.88	0.00
	03	12.22(8.76,15.67)	3.69(1.70,5.68)	4.19	0.00
	04	13.17(9.59,16.76)	4.25(2.17,6.32)	4.22	0.00
	05	14.12(10.42,17.81)	4.74(2.59,6.88)	4.30	0.00
	06	14.44(10.64,18.23)	5.16(2.96,7.36)	4.15	0.00
	07	14.61(10.72,18.49)	5.25(3.00,7.50)	4.09	0.00
Hospital cost	0	7551.77(3690.47,11413.06)	2285.95(319.38,4252.53)	2.38	0.02
	1	8896.91(5198.52,12595.31)	1404.18(-484.31,3292.68)	3.54	0.00
	2	10394.92(6709.88,14079.96)	1969.57(84.71,3854.43)	3.99	0.00
	3	10182.76(6454.93,13910.59)	2083.46(177.27,3989.64)	3.79	0.00
	4	10399.13(6637.24,14161.01)	1701.96(-223.31,3627.24)	4.03	0.00
	5	11481.13(7732.72,15229.53)	2964.66(1035.60,4893.72)	3.96	0.00
	6	9780.31(6013.96,13546.66)	2541.75(605.51,4477.99)	3.35	0.00
	7	8091.29(4302.83,11879.74)	1727.77(-218.30,3673.84)	2.93	0.00
	01	9602.97(5531.21,13674.73)	2113.22(35.34,4191.11)	3.21	0.00
	02	11732.29(7460.91,16003.67)	2431.84(248.50,4615.19)	3.80	0.00
	03	13319.76(8857.37,17782.15)	2751.10(467.63,5034.58)	4.13	0.00
	04	14681.54(10051.89,19311.20)	2910.27(538.58,5281.96)	4.44	0.00
	05	16307.31(11540.20,21074.42)	3370.11(924.34,5815.87)	4.73	0.00
	06	17106.56(12217.99,21995.13)	3666.58(1156.70,6176.47)	4.79	0.00
	07	17524.65(12520.51,22528.79)	3739.34(1168.71,6309.96)	4.80	0.00

Table S9 The NO_2 effect of admissions, LOS and hospital cost for RD-DM on age group with different lag day.

Outcome	Lag day	Younger adult Increase (95% CI)	The elderly Increase (95% CI)	Z Value	P Value
Admissions	0	0.22(-3.41,4.00)	7.69(4.81,10.64)	3.07	0.00
	1	0.12(-3.41,3.79)	8.63(5.83,11.51)	3.60	0.00
	2	0.74(-2.84,4.44)	10.54(7.70,13.46)	4.09	0.00
	3	-0.06(-3.62,3.63)	11.33(8.46,14.28)	4.73	0.00
	4	1.64(-2.06,5.48)	10.63(7.71,13.62)	3.63	0.00
	5	2.83(-0.91,6.72)	10.31(7.37,13.33)	3.00	0.00
	6	4.69(0.93,8.60)	7.55(4.68,10.51)	1.16	0.25
	7	3.42(-0.27,7.24)	7.37(4.51,10.30)	1.62	0.10
	01	0.18(-3.68,4.19)	9.51(6.42,12.68)	3.59	0.00
	02	0.45(-3.63,4.70)	11.76(8.46,15.17)	4.09	0.00
	03	0.35(-3.91,4.80)	13.85(10.34,17.47)	4.63	0.00
	04	0.84(-3.61,5.49)	15.28(11.60,19.08)	4.72	0.00
	05	1.52(-3.10,6.37)	16.43(12.60,20.40)	4.68	0.00
	06	2.69(-2.11,7.73)	16.74(12.78,20.83)	4.26	0.00
	07	3.24(-1.69,8.43)	17.11(13.05,21.33)	4.09	0.00
LOS	0	0.22(-1.24,1.69)	9.67(6.43,12.91)	5.20	0.00
	1	0.56(-0.85,1.96)	9.43(6.32,12.53)	5.09	0.00
	2	0.54(-0.86,1.94)	11.66(8.57,14.74)	6.42	0.00
	3	0.57(-0.85,1.98)	12.08(8.96,15.20)	6.58	0.00
	4	1.25(-0.19,2.68)	10.68(7.52,13.84)	5.33	0.00
	5	1.51(0.07,2.94)	10.91(7.74,14.07)	5.30	0.00
	6	2.17(0.74,3.61)	8.47(5.28,11.65)	3.53	0.00
	7	1.45(0.01,2.89)	7.29(4.09,10.49)	3.26	0.00
	01	0.46(-1.09,2.01)	11.10(7.68,14.52)	5.56	0.00
	02	0.58(-1.04,2.20)	13.39(9.81,16.98)	6.39	0.00
	03	0.68(-1.01,2.38)	15.37(11.63,19.10)	7.01	0.00
	04	0.97(-0.80,2.73)	16.59(12.71,20.47)	7.19	0.00
	05	1.29(-0.53,3.10)	17.73(13.74,21.73)	7.35	0.00
	06	1.74(-0.13,3.61)	18.06(13.96,22.16)	7.10	0.00
	07	1.91(0.00,3.82)	18.19(13.99,22.39)	6.92	0.00
Hospital cost	0	583.62(-1199.1,2366.33)	9386.15(5308.50,13463.81)	3.88	0.00
	1	362.95(-1349.51,2075.41)	10063.81(6156.67,13970.96)	4.46	0.00
	2	-256.48(-1966.06,1453.09)	12747.27(8862.23,16632.30)	6.00	0.00
	3	44.92(-1684.18,1774.02)	12334.20(8401.10,16267.30)	5.61	0.00
	4	1127.91(-617.23,2873.04)	11052.14(7071.68,15032.60)	4.48	0.00
	5	1481.49(-268.05,3231.03)	13111.89(9137.91,17085.86)	5.25	0.00
	6	2205.32(451.28,3959.36)	10217.40(6208.64,14226.16)	3.59	0.00
	7	1227.57(-535.66,2990.8)	8803.91(4775.83,12831.99)	3.38	0.00
	01	540.65(-1340.16,2421.46)	11320.92(7020.24,15621.59)	4.50	0.00
	02	280.91(-1692.99,2254.80)	14042.00(9534.28,18549.72)	5.48	0.00
	03	247.85(-1814.24,2309.93)	15982.86(11275.55,20690.18)	6.00	0.00
	04	545.71(-1593.73,2685.16)	17191.84(12305.93,22077.76)	6.12	0.00
	05	909.46(-1295.40,3114.32)	18897.63(13865.56,23929.69)	6.42	0.00
	06	1368.08(-893.24,3629.40)	19525.37(14360.81,24689.92)	6.31	0.00
	07	1517.56(-796.71,3831.82)	19887.91(14597.23,25178.59)	6.24	0.00

Table S10 The CO effect of admissions, LOS and hospital cost for RD-DM on gender group with different lag day.

Outcome	Lag day	Male	Female	Z Value	P Value
		Increase (95% CI)	Increase (95% CI)		
Admissions	0	12.50(7.21,18.06)	6.94(0.49,13.80)	1.26	0.21
	1	13.85(8.62,19.34)	5.74(-0.56,12.44)	1.87	0.06
	2	13.39(8.22,18.81)	10.43(3.98,17.28)	0.68	0.50
	3	11.20(5.96,16.69)	8.67(2.14,15.62)	0.57	0.57
	4	13.36(7.91,19.09)	11.38(4.53,18.69)	0.43	0.67
	5	11.69(6.31,17.34)	10.15(3.44,17.29)	0.34	0.73
	6	7.83(2.71,13.21)	6.89(0.54,13.65)	0.22	0.83
	7	9.97(4.70,15.49)	2.46(-3.79,9.12)	1.74	0.08
	01	17.25(11.08,23.77)	8.18(0.86,16.03)	1.78	0.07
	02	20.63(13.8,27.87)	11.87(3.76,20.63)	1.55	0.12
	03	22.62(15.23,30.49)	14.00(5.18,23.56)	1.40	0.16
	04	25.41(17.44,33.92)	16.93(7.37,27.34)	1.27	0.20
	05	27.45(18.98,36.53)	19.11(8.95,30.23)	1.18	0.24
	06	27.95(19.15,37.39)	20.03(9.45,31.64)	1.07	0.28
	07	29.36(20.18,39.25)	19.37(8.51,31.31)	1.31	0.19
LOS	0	14.41(8.94,19.88)	3.53(0.40,6.66)	3.38	0.00
	1	12.02(6.72,17.33)	3.89(0.86,6.91)	2.61	0.01
	2	12.31(7.01,17.61)	6.46(3.43,9.49)	1.88	0.06
	3	11.80(6.44,17.15)	5.06(2.00,8.12)	2.14	0.03
	4	13.19(7.79,18.58)	5.47(2.39,8.54)	2.44	0.01
	5	11.66(6.25,17.07)	4.27(1.18,7.36)	2.32	0.02
	6	4.60(-0.84,10.05)	4.01(0.91,7.10)	0.19	0.85
	7	8.80(3.36,14.25)	1.36(-1.76,4.48)	2.32	0.02
	01	16.79(10.72,22.85)	4.71(1.24,8.18)	3.39	0.00
	02	19.16(12.65,25.67)	6.86(3.14,10.58)	3.22	0.00
	03	21.13(14.23,28.03)	7.97(4.01,11.93)	3.24	0.00
	04	23.43(16.20,30.66)	9.13(4.96,13.31)	3.36	0.00
	05	24.94(17.43,32.46)	9.68(5.33,14.03)	3.44	0.00
	06	24.20(16.42,31.98)	10.12(5.62,14.62)	3.07	0.00
	07	24.89(16.88,32.90)	9.72(5.09,14.35)	3.21	0.00
Hospital cost	0	17715.31(10669.75,24760.87)	2557.69(-1041.80,6157.18)	3.76	0.00
	1	13467.22(6624.29,20310.15)	1624.81(-1859.45,5109.07)	3.02	0.00
	2	15485.07(8663.18,22306.97)	4142.28(659.43,7625.14)	2.90	0.00
	3	15024.40(8131.01,21917.80)	3505.90(-17.59,7029.39)	2.92	0.00
	4	16195.60(9245.79,23145.41)	2360.78(-1197.81,5919.38)	3.47	0.00
	5	16589.71(9655.09,23524.33)	2901.55(-668.41,6471.50)	3.44	0.00
	6	10740.02(3744.71,17735.33)	2477.64(-1102.28,6057.57)	2.06	0.04
	7	12833.32(5832.89,19833.76)	1003.80(-2590.68,4598.29)	2.95	0.00
	01	19904.58(12093.56,27715.61)	2630.18(-1363.38,6623.74)	3.86	0.00
	02	23289.06(14922.31,31655.80)	4110.95(-176.73,8398.63)	4.00	0.00
	03	26045.67(17181.83,34909.51)	4930.11(380.47,9479.75)	4.15	0.00
	04	28985.21(19702.29,38268.13)	5208.45(431.54,9985.37)	4.46	0.00
	05	31694.88(22063.88,41325.87)	5647.13(677.16,10617.10)	4.71	0.00
	06	32345.76(22398.61,42292.91)	5924.24(785.96,11062.53)	4.63	0.00
	07	33694.25(23462.88,43925.63)	5756.40(459.75,11053.05)	4.75	0.00

Table S11 The CO effect of admissions, LOS and hospital cost for RD-DM on age group with different lag day.

Outcome	Lag day	Younger adult	The elderly	Z Value	P Value
		Increase (95% CI)	Increase (95% CI)		
Admissions	0	4.66(-2.06,11.84)	13.98(8.71,19.50)	2.05	0.04
	1	5.29(-1.33,12.36)	14.16(8.98,19.59)	1.99	0.05
	2	6.33(-0.31,13.41)	15.85(10.68,21.26)	2.13	0.03
	3	2.27(-4.38,9.39)	14.72(9.49,20.21)	2.75	0.01
	4	9.67(2.45,17.40)	14.25(8.86,19.90)	0.96	0.34
	5	11.68(4.48,19.37)	10.95(5.67,16.49)	0.16	0.88
	6	7.55(0.79,14.75)	7.72(2.65,13.03)	0.04	0.97
	7	5.60(-1.17,12.84)	8.17(3.05,13.55)	0.57	0.57
	01	6.37(-1.24,14.56)	18.45(12.30,24.94)	2.31	0.02
	02	8.16(-0.12,17.12)	23.06(16.20,30.34)	2.58	0.01
	03	7.94(-0.87,17.54)	26.49(18.99,34.47)	2.96	0.00
	04	10.63(1.1,21.07)	29.33(21.25,37.96)	2.76	0.01
	05	13.74(3.52,24.97)	30.70(22.17,39.83)	2.35	0.02
	06	15.40(4.69,27.20)	30.83(21.97,40.33)	2.05	0.04
	07	16.02(4.94,28.28)	31.40(22.21,41.29)	1.97	0.05
LOS	0	2.50(-0.18,5.18)	15.61(9.67,21.54)	3.95	0.00
	1	2.71(0.12,5.31)	13.43(7.68,19.18)	3.33	0.00
	2	2.96(0.35,5.56)	16.21(10.47,21.95)	4.12	0.00
	3	1.68(-0.96,4.33)	15.68(9.88,21.47)	4.31	0.00
	4	4.26(1.60,6.93)	15.00(9.15,20.85)	3.28	0.00
	5	4.72(2.06,7.39)	11.80(5.92,17.68)	2.15	0.03
	6	2.87(0.20,5.54)	6.26(0.35,12.16)	1.02	0.31
	7	2.57(-0.11,5.25)	8.21(2.30,14.13)	1.70	0.09
	01	3.32(0.35,6.30)	18.43(11.85,25.01)	4.10	0.00
	02	4.06(0.86,7.26)	22.37(15.31,29.42)	4.63	0.00
	03	4.17(0.76,7.57)	25.44(17.96,32.91)	5.08	0.00
	04	5.26(1.68,8.84)	27.92(20.08,35.75)	5.16	0.00
	05	6.33(2.60,10.05)	29.04(20.89,37.19)	4.97	0.00
	06	6.73(2.88,10.58)	28.43(20.00,36.86)	4.59	0.00
	07	6.97(3.00,10.93)	28.63(19.94,37.32)	4.45	0.00
Hospital cost	0	4069.12(795.82,7342.43)	16104.85(8638.21,23571.48)	2.89	0.00
	1	2374.32(-796.08,5544.71)	12694.02(5452.79,19935.25)	2.56	0.01
	2	1247.06(-1926.83,4420.95)	18350.17(11142.17,25558.17)	4.26	0.00
	3	958.51(-2253.18,4170.20)	17526.47(10233.15,24819.79)	4.07	0.00
	4	4249.07(1013.15,7484.99)	14245.79(6861.23,21630.35)	2.43	0.02
	5	5258.85(2016.35,8501.36)	13991.83(6582.99,21400.67)	2.12	0.03
	6	3738.94(482.30,6995.58)	9367.95(1922.01,16813.89)	1.36	0.17
	7	2639.98(-630.91,5910.88)	11136.20(3676.20,18596.19)	2.04	0.04
	01	4058.40(432.72,7684.07)	18351.05(10069.76,26632.34)	3.10	0.00
	02	3752.15(-138.12,7642.43)	23478.01(14611.88,32344.13)	3.99	0.00
	03	3572.66(-552.77,7698.09)	27150.56(17752.13,36548.99)	4.50	0.00
	04	4686.69(362.68,9010.70)	29146.63(19281.58,39011.67)	4.45	0.00
	05	6019.09(1514.24,10523.94)	30924.02(20662.32,41185.72)	4.36	0.00
	06	6696.92(2039.68,11354.15)	31175.23(20560.39,41790.07)	4.14	0.00
	07	6947.93(2150.81,11745.05)	32071.98(21132.73,43011.22)	4.12	0.00

Table S12 The O_3 effect of admissions, LOS and hospital cost for RD-DM on gender group with different lag day.

Outcome	Lag day	Male	Female	Z Value	P Value
		Increase (95% CI)	Increase (95% CI)		
Admissions	0	-1.67(-2.79,-0.54)	-2.20(-3.59,-0.79)	0.58	0.56
	1	-2.27(-3.33,-1.21)	-2.11(-3.42,-0.78)	0.19	0.85
	2	-1.74(-2.72,-0.75)	-1.54(-2.76,-0.31)	0.25	0.80
	3	-1.38(-2.32,-0.42)	-0.97(-2.15,0.22)	0.52	0.60
	4	-1.78(-2.71,-0.84)	-1.17(-2.34,0.00)	0.80	0.43
	5	-1.81(-2.72,-0.90)	-1.13(-2.27,0.02)	0.91	0.36
	6	-1.93(-2.82,-1.03)	-0.98(-2.10,0.15)	1.29	0.20
	7	-2.22(-3.10,-1.33)	-1.67(-2.78,-0.55)	0.75	0.45
	01	-2.53(-3.74,-1.30)	-2.72(-4.24,-1.19)	0.19	0.85
	02	-2.71(-3.95,-1.45)	-2.74(-4.28,-1.16)	0.02	0.98
	03	-2.69(-3.94,-1.42)	-2.51(-4.08,-0.92)	0.17	0.86
	04	-2.85(-4.11,-1.57)	-2.47(-4.04,-0.87)	0.37	0.71
	05	-2.98(-4.24,-1.71)	-2.44(-4.02,-0.83)	0.53	0.60
	06	-3.12(-4.37,-1.84)	-2.37(-3.95,-0.77)	0.72	0.47
	07	-3.31(-4.56,-2.03)	-2.52(-4.10,-0.91)	0.76	0.45
LOS	0	-1.87(-3.02,-0.72)	-0.95(-1.60,-0.30)	1.37	0.17
	1	-2.61(-3.69,-1.53)	-0.79(-1.40,-0.17)	2.86	0.00
	2	-1.25(-2.25,-0.25)	-0.73(-1.30,-0.16)	0.89	0.38
	3	-1.20(-2.16,-0.23)	-0.48(-1.03,0.07)	1.27	0.20
	4	-1.33(-2.28,-0.38)	-0.61(-1.16,-0.07)	1.28	0.20
	5	-1.27(-2.21,-0.33)	-0.63(-1.17,-0.10)	1.16	0.25
	6	-1.49(-2.41,-0.57)	-0.47(-1.00,0.06)	1.89	0.06
	7	-1.92(-2.83,-1.00)	-0.69(-1.21,-0.17)	2.28	0.02
	01	-2.88(-4.13,-1.62)	-1.09(-1.80,-0.38)	2.43	0.02
	02	-2.66(-3.94,-1.38)	-1.16(-1.89,-0.43)	1.99	0.05
	03	-2.58(-3.87,-1.28)	-1.10(-1.84,-0.37)	1.94	0.05
	04	-2.60(-3.91,-1.30)	-1.13(-1.87,-0.38)	1.93	0.05
	05	-2.61(-3.92,-1.3)	-1.16(-1.90,-0.41)	1.88	0.06
	06	-2.68(-4.00,-1.37)	-1.13(-1.88,-0.38)	2.01	0.04
	07	-2.86(-4.18,-1.55)	-1.18(-1.93,-0.43)	2.17	0.03
Hospital cost	0	-1991.93(-3477.44,-506.42)	-815.00(-1562.22,-67.79)	1.39	0.17
	1	-2988.84(-4388.53,-1589.15)	-884.27(-1596.16,-172.39)	2.63	0.01
	2	-1721.74(-3014.30,-429.17)	-785.33(-1442.53,-128.13)	1.27	0.21
	3	-1685.19(-2930.41,-439.97)	-492.73(-1125.16,139.70)	1.67	0.09
	4	-1530.68(-2760.39,-300.97)	-818.65(-1442.18,-195.13)	1.01	0.31
	5	-1854.24(-3057.52,-650.95)	-613.72(-1227.47,0.03)	1.80	0.07
	6	-1792.29(-2979.45,-605.14)	-593.82(-1199.13,11.49)	1.76	0.08
	7	-2600.38(-3773.56,-1427.19)	-763.86(-1363.86,-163.85)	2.73	0.01
	01	-3198.07(-4818.72,-1577.42)	-1075.45(-1894.63,-256.27)	2.29	0.02
	02	-3145.52(-4798.61,-1492.43)	-1183.45(-2024.08,-342.82)	2.07	0.04
	03	-3139.65(-4807.06,-1472.25)	-1129.02(-1979.09,-278.95)	2.11	0.04
	04	-3122.80(-4804.16,-1441.44)	-1226.97(-2083.96,-369.97)	1.97	0.05
	05	-3231.00(-4919.50,-1542.49)	-1233.32(-2093.94,-372.70)	2.07	0.04
	06	-3306.49(-4998.83,-1614.16)	-1234.50(-2096.87,-372.12)	2.14	0.03
	07	-3588.57(-5284.72,-1892.41)	-1286.70(-2151.12,-422.28)	2.37	0.02

Table S13 The O_3 effect of admissions, LOS and hospital cost for RD-DM on age group with different lag day.

Outcome	Lag day	Younger adult	The elderly	Z Value	P Value
		Increase (95% CI)	Increase (95% CI)		
Admissions	0	-1.66(-3.08,-0.21)	-2.11(-3.24,-0.96)	0.48	0.63
	1	-1.44(-2.78,-0.07)	-2.70(-3.76,-1.63)	1.44	0.15
	2	-1.12(-2.37,0.15)	-2.01(-2.99,-1.02)	1.09	0.28
	3	-0.75(-1.97,0.48)	-1.55(-2.50,-0.60)	1.02	0.31
	4	-0.80(-2.01,0.42)	-2.05(-2.98,-1.11)	1.60	0.11
	5	-0.48(-1.66,0.71)	-2.28(-3.19,-1.37)	2.37	0.02
	6	-0.68(-1.83,0.49)	-2.17(-3.07,-1.27)	2.01	0.04
	7	-0.80(-1.95,0.36)	-2.80(-3.68,-1.91)	2.70	0.01
	01	-1.96(-3.51,-0.39)	-3.08(-4.31,-1.84)	1.10	0.27
	02	-1.95(-3.54,-0.33)	-3.21(-4.45,-1.95)	1.22	0.22
	03	-1.80(-3.41,-0.16)	-3.13(-4.38,-1.86)	1.27	0.21
	04	-1.75(-3.37,-0.10)	-3.30(-4.56,-2.02)	1.47	0.14
	05	-1.62(-3.25,0.04)	-3.51(-4.77,-2.24)	1.79	0.07
	06	-1.58(-3.21,0.07)	-3.64(-4.89,-2.36)	1.94	0.05
	07	-1.60(-3.24,0.06)	-3.91(-5.16,-2.64)	2.18	0.03
LOS	0	-0.67(-1.23,-0.12)	-2.32(-3.57,-1.07)	2.36	0.02
	1	-0.58(-1.11,-0.05)	-2.91(-4.09,-1.73)	3.55	0.00
	2	-0.41(-0.90,0.07)	-1.60(-2.69,-0.51)	1.94	0.05
	3	-0.37(-0.84,0.10)	-1.35(-2.40,-0.30)	1.67	0.09
	4	-0.48(-0.94,-0.01)	-1.51(-2.55,-0.48)	1.79	0.07
	5	-0.32(-0.78,0.14)	-1.64(-2.66,-0.63)	2.33	0.02
	6	-0.27(-0.72,0.18)	-1.75(-2.76,-0.75)	2.66	0.01
	7	-0.30(-0.74,0.15)	-2.38(-3.37,-1.39)	3.76	0.00
	01	-0.79(-1.40,-0.18)	-3.34(-4.71,-1.98)	3.35	0.00
	02	-0.78(-1.40,-0.15)	-3.18(-4.57,-1.78)	3.08	0.00
	03	-0.75(-1.38,-0.12)	-3.01(-4.42,-1.61)	2.88	0.00
	04	-0.79(-1.42,-0.15)	-3.01(-4.42,-1.59)	2.80	0.01
	05	-0.76(-1.40,-0.12)	-3.07(-4.49,-1.64)	2.90	0.00
	06	-0.73(-1.37,-0.09)	-3.16(-4.58,-1.73)	3.04	0.00
	07	-0.72(-1.36,-0.08)	-3.40(-4.83,-1.97)	3.35	0.00
Hospital cost	0	-542.54(-1224.31,139.24)	-2354.99(-3928.71,-781.27)	2.07	0.04
	1	-477.03(-1126.85,172.79)	-3443.76(-4923.14,-1964.3	3.60	0.00
	2	-328.11(-927.73,271.52)	-2195.09(-3560.42,-829.77)	2.45	0.01
	3	-342.99(-919.27,233.28)	-1850.67(-3166.79,-534.54)	2.06	0.04
	4	-617.31(-1185.49,-49.12)	-1746.47(-3045.80,-447.14)	1.56	0.12
	5	-381.38(-940.05,177.28)	-2109.94(-3384.78,-835.09)	2.43	0.01
	6	-299.40(-850.21,251.40)	-2114.53(-3371.88,-857.18)	2.59	0.01
	7	-408.11(-954.11,137.88)	-2983.49(-4225.70,-1741.2	3.72	0.00
	01	-641.20(-1388.14,105.75)	-3744.40(-5459.00,-2029.7	3.25	0.00
	02	-627.44(-1393.44,138.55)	-3774.37(-5522.46,-2026.2	3.23	0.00
	03	-634.51(-1408.25,139.23)	-3682.71(-5445.89,-1919.5	3.10	0.00
	04	-749.47(-1528.94,29.99)	-3636.75(-5414.59,-1858.9	2.92	0.00
	05	-753.76(-1536.03,28.52)	-3743.12(-5528.48,-1957.7	3.01	0.00
	06	-735.53(-1518.99,47.93)	-3836.25(-5625.63,-2046.8	3.11	0.00
	07	-754.12(-1539.05,30.81)	-4151.44(-5944.75,-2358.1	3.40	0.00

Table S14 Percent change (95% CI）in hospital admissions for RD-DM associated with one unit increase in $PM_{2.5}$, PM_{10}, SO_2, NO_2, CO and O_3 along different lag days using single-pollutant and two-pollutant models.

	**Lag0**	**Lag1**	**Lag2**	**Lag3**	**Lag4**	**Lag5**	**Lag6**	**Lag7**
**$PM_{2.5}$**	1.31(-0.55,3.20)	**1.87(0.00,3.78)**	**3.38(1.45,5.34)**	**3.20(1.26,5.18)**	**4.15(2.19,6.16)**	**4.82(2.83,6.85)**	**3.85(1.85,5.90)**	**3.48(1.50,5.50)**
+SO_2	-0.56(-2.54,1.45)	1.10(-0.78,3.02)	**3.06(1.14,5.02)**	**3.02(1.09,4.99)**	**3.99(2.03,5.99)**	**4.50(2.52,6.53)**	**3.32(1.32,5.35)**	**2.92(0.94,4.93)**
+NO_2	-1.57(-3.89,0.81)	0.41(-1.63,2.48)	**2.60(0.62,4.62)**	**2.56(0.58,4.57)**	**3.59(1.59,5.62)**	**4.28(2.26,6.34)**	**3.36(1.34,5.41)**	**3.07(1.08,5.10)**
+CO	-0.91(-2.97,1.20)	0.50(-1.46,2.49)	**2.59(0.65,4.58)**	**2.63(0.68,4.60)**	**3.63(1.67,5.64)**	**4.23(2.23,6.27)**	**3.14(1.12,5.19)**	**2.80(0.82,4.83)**
+O_3	**2.85(0.87,4.88)**	**2.61(0.70,4.55)**	**3.93(1.99,5.91)**	**3.77(1.81,5.76)**	**4.68(2.71,6.70)**	**5.26(3.27,7.29)**	**4.16(2.16,6.20)**	**3.67(1.69,5.68)**
**PM_{10}**	**1.74(0.57,2.93)**	**2.07(0.89,3.26)**	**3.07(1.86,4.28)**	**2.78(1.57,4.01)**	**3.12(1.89,4.37)**	**3.04(1.81,4.29)**	**2.53(1.30,3.77)**	**2.42(1.19,3.65)**
+SO_2	0.47(-0.83,1.79)	**1.51(0.31,2.72)**	**2.77(1.57,3.99)**	**2.54(1.33,3.77)**	**2.90(1.67,4.14)**	**2.74(1.50,3.98)**	**2.10(0.88,3.35)**	**2.02(0.80,3.26)**
+NO_2	0.11(-1.60,1.85)	1.23(-0.11,2.59)	**2.59(1.33,3.86)**	**2.36(1.12,3.62)**	**2.75(1.49,4.03)**	**2.66(1.40,3.94)**	**2.18(0.94,3.44)**	**2.11(0.88,3.36)**
+CO	0.35(-1.04,1.76)	1.25(-0.01,2.52)	**2.58(1.35,3.82)**	**2.42(1.20,3.65)**	**2.80(1.57,4.05)**	**2.66(1.42,3.92)**	**2.10(0.86,3.35)**	**2.05(0.82,3.29)**
+O_3	**2.41(1.21,3.63)**	**2.30(1.12,3.49)**	**3.21(2.01,4.42)**	**2.95(1.75,4.18)**	**3.32(2.09,4.56)**	**3.18(1.95,4.43)**	**2.63(1.40,3.86)**	**2.46(1.25,3.69)**
**SO_2**	**3.96(2.40,5.55)**	**2.48(0.93,4.06)**	**2.81(1.30,4.34)**	**3.83(2.31,5.36)**	**4.90(3.36,6.45)**	**3.77(2.22,5.34)**	**2.25(0.73,3.78)**	**2.19(0.65,3.74)**
+$PM_{2.5}$	**4.15(2.46,5.86)**	**2.36(0.78,3.96)**	**2.75(1.24,4.29)**	**3.79(2.27,5.33)**	**4.86(3.32,6.42)**	**3.70(2.15,5.28)**	**2.17(0.65,3.71)**	**2.11(0.57,3.67)**
+PM_{10}	**3.67(1.91,5.46)**	**2.03(0.43,3.66)**	**2.62(1.09,4.16)**	**3.70(2.18,5.24)**	**4.76(3.22,6.33)**	**3.58(2.02,5.16)**	**2.02(0.50,3.57)**	**1.94(0.39,3.50)**
+NO_2	**3.27(1.58,4.98)**	**1.93(0.35,3.54)**	**2.59(1.08,4.13)**	**3.72(2.20,5.26)**	**4.86(3.32,6.42)**	**3.62(2.07,5.19)**	**2.05(0.54,3.59)**	**1.99(0.46,3.55)**
+CO	**2.85(1.01,4.73)**	**1.68(0.08,3.30)**	**2.42(0.90,3.95)**	**3.64(2.13,5.17)**	**4.70(3.17,6.25)**	**3.48(1.93,5.05)**	**1.91(0.40,3.44)**	**1.98(0.45,3.53)**
+O_3	**3.88(2.34,5.45)**	**2.20(0.65,3.77)**	**2.57(1.06,4.09)**	**3.68(2.18,5.21)**	**4.72(3.20,6.27)**	**3.60(2.06,5.16)**	**2.02(0.51,3.55)**	**2.01(0.48,3.56)**
**NO_2**	**4.82(2.35,7.35)**	**5.45(3.04,7.92)**	**6.98(4.53,9.48)**	**7.25(4.78,9.79)**	**7.51(4.98,10.10)**	**7.63(5.09,10.24)**	**6.41(3.92,8.96)**	**5.77(3.31,8.29)**
+$PM_{2.5}$	**6.16(2.97,9.46)**	**5.87(3.17,8.65)**	**7.00(4.47,9.60)**	**7.17(4.65,9.74)**	**7.41(4.84,10.03)**	**7.53(4.96,10.17)**	**6.30(3.79,8.87)**	**5.68(3.21,8.21)**

+PM_{10}	**4.64(1.00,8.41)**	**5.00(2.10,7.97)**	**6.50(3.90,9.17)**	**6.76(4.21,9.38)**	**7.02(4.41,9.68)**	**7.16(4.55,9.83)**	**5.94(3.40,8.54)**	**5.34(2.85,7.90)**
+SO_2	**2.86(0.23,5.55)**	**4.34(1.89,6.85)**	**6.50(4.05,9.00)**	**6.96(4.49,9.48)**	**7.13(4.61,9.72)**	**7.24(4.70,9.83)**	**5.86(3.38,8.40)**	**5.33(2.88,7.84)**
+CO	1.93(-1.26,5.22)	**3.61(0.88,6.42)**	**5.80(3.25,8.42)**	**6.27(3.75,8.86)**	**6.53(3.95,9.17)**	**6.65(4.06,9.30)**	**5.33(2.78,7.93)**	**4.82(2.32,7.37)**
+O3	**5.19(2.72,7.71)**	**5.28(2.89,7.72)**	**6.58(4.16,9.07)**	**6.81(4.37,9.32)**	**6.99(4.50,9.55)**	**7.12(4.60,9.69)**	**5.93(3.46,8.47)**	**5.38(2.93,7.89)**
**CO**	**10.23(5.69,14.96)**	**10.63(6.17,15.28)**	**12.21(7.73,16.87)**	**10.12(5.59,14.84)**	**12.52(7.78,17.46)**	**10.85(6.21,15.69)**	**7.20(2.79,11.79)**	**6.81(2.36,11.46)**
+$PM_{2.5}$	**11.33(6.14,16.77)**	**10.47(5.89,15.25)**	**12.03(7.51,16.73)**	**9.95(5.40,14.71)**	**12.33(7.57,17.30)**	**10.65(5.98,15.52)**	**7.03(2.61,11.64)**	**6.68(2.22,11.33)**
+PM_{10}	**9.43(3.97,15.17)**	**9.53(4.87,14.40)**	**11.47(6.93,16.21)**	**9.51(4.93,14.29)**	**11.85(7.07,16.83)**	**10.19(5.51,15.08)**	**6.69(2.25,11.32)**	**6.33(1.86,11.00)**
+SO_2	**5.58(0.41,11.02)**	**8.35(3.82,13.08)**	**10.92(6.44,15.58)**	**9.29(4.77,14.00)**	**11.31(6.62,16.20)**	**9.67(5.07,14.48)**	**5.85(1.47,10.41)**	**5.70(1.27,10.33)**
+NO_2	**7.79(1.85,14.07)**	**8.54(3.73,13.57)**	**10.72(6.12,15.52)**	**8.78(4.17,13.59)**	**11.30(6.52,16.31)**	**9.66(4.97,14.57)**	**6.13(1.68,10.77)**	**5.92(1.44,10.60)**
+O_3	**9.68(5.21,14.35)**	**9.91(5.48,14.52)**	**11.27(6.85,15.87)**	**9.24(4.77,13.90)**	**11.41(6.76,16.27)**	**9.97(5.37,14.76)**	**6.41(2.04,10.97)**	**6.18(1.76,10.79)**
**O_3**	**-1.87(-2.84,-0.90)**	**-2.21(-3.12,-1.30)**	**-1.66(-2.50,-0.81)**	**-1.21(-2.03,-0.39)**	**-1.54(-2.34,-0.73)**	**-1.54(-2.32,-0.75)**	**-1.55(-2.32,-0.77)**	**-1.99(-2.76,-1.23)**
+$PM_{2.5}$	**-2.36(-3.38,-1.34)**	**-2.61(-3.55,-1.66)**	**-1.84(-2.69,-0.97)**	**-1.28(-2.10,-0.46)**	**-1.59(-2.40,-0.78)**	**-1.58(-2.36,-0.79)**	**-1.60(-2.37,-0.82)**	**-2.03(-2.80,-1.26)**
+PM_{10}	**-2.34(-3.32,-1.35)**	**-2.62(-3.54,-1.69)**	**-1.82(-2.66,-0.96)**	**-1.25(-2.07,-0.43)**	**-1.55(-2.35,-0.74)**	**-1.53(-2.31,-0.74)**	**-1.55(-2.32,-0.77)**	**-1.98(-2.75,-1.21)**
+SO_2	**-1.81(-2.77,-0.85)**	**-2.07(-2.98,-1.16)**	**-1.56(-2.40,-0.71)**	**-1.13(-1.94,-0.31)**	**-1.40(-2.20,-0.59)**	**-1.41(-2.19,-0.62)**	**-1.42(-2.19,-0.64)**	**-1.86(-2.62,-1.09)**
+NO_2	**-2.03(-2.99,-1.07)**	**-2.41(-3.31,-1.49)**	**-1.64(-2.48,-0.79)**	**-1.11(-1.93,-0.28)**	**-1.39(-2.20,-0.58)**	**-1.40(-2.19,-0.60)**	**-1.42(-2.20,-0.64)**	**-1.87(-2.64,-1.09)**
+CO	**-1.75(-2.70,-0.78)**	**-2.08(-2.98,-1.17)**	**-1.54(-2.38,-0.69)**	**-1.05(-1.87,-0.23)**	**-1.35(-2.16,-0.54)**	**-1.36(-2.15,-0.57)**	**-1.39(-2.17,-0.61)**	**-1.89(-2.65,-1.11)**

Notes: Bold fonts indicates the effect was statistically significant (P<0.05).

Table S15 Absolute increase (95% CI）in LOS (length of hospital stay) for RD-DM associated with one unit increase in$PM_{2.5}$, PM_{10}, SO_2, NO_2, CO and O_3 along different lag days using single-pollutant and two-pollutant models.

	Lag0	Lag1	Lag2	Lag3	Lag4	Lag5	Lag6	Lag7
**$PM_{2.5}$**	**3.77(0.96,6.58)**	**3.79(0.98,6.61)**	**5.87(3.04,8.70)**	**5.01(2.15,7.87)**	**6.34(3.46,9.23)**	**7.95(5.06,10.84)**	**6.07(3.15,8.98)**	**5.69(2.76,8.63)**
+SO_2	1.45(-1.57,4.47)	2.80(-0.04,5.63)	**5.42(2.60,8.24)**	**4.69(1.84,7.54)**	**5.97(3.10,8.84)**	**7.47(4.58,10.35)**	**5.45(2.54,8.36)**	**5.04(2.11,7.98)**
+NO_2	-1.36(-4.91,2.18)	0.87(-2.20,3.93)	**4.25(1.34,7.16)**	**3.63(0.72,6.53)**	**5.12(2.20,8.03)**	**6.81(3.88,9.73)**	**5.03(2.10,7.96)**	**4.82(1.89,7.76)**
+CO	0.36(-2.81,3.53)	1.55(-1.41,4.51)	**4.60(1.73,7.46)**	**4.06(1.19,6.93)**	**5.41(2.52,8.31)**	**6.95(4.04,9.86)**	**4.97(2.04,7.91)**	**4.65(1.70,7.60)**
+O_3	**6.66(3.66,9.65)**	**5.02(2.17,7.87)**	**6.80(3.96,9.65)**	**5.99(3.11,8.86)**	**7.24(4.35,10.14)**	**8.67(5.78,11.56)**	**6.64(3.73,9.56)**	**6.09(3.16,9.01)**
**PM_{10}**	**3.64(1.87,5.41)**	**3.65(1.89,5.41)**	**5.00(3.24,6.77)**	**4.25(2.46,6.04)**	**4.75(2.95,6.55)**	**5.15(3.35,6.96)**	**4.10(2.27,5.92)**	**3.80(1.97,5.63)**
+SO_2	**2.17(0.20,4.14)**	**2.94(1.15,4.73)**	**4.60(2.83,6.37)**	**3.89(2.10,5.68)**	**4.35(2.55,6.15)**	**4.73(2.91,6.54)**	**3.60(1.77,5.43)**	**3.34(1.50,5.17)**
+NO_2	0.44(-2.12,3.00)	1.83(-0.15,3.82)	**3.92(2.07,5.77)**	**3.31(1.48,5.14)**	**3.91(2.08,5.74)**	**4.34(2.50,6.19)**	**3.36(1.51,5.20)**	**3.15(1.30,4.99)**
+CO	1.57(-0.52,3.66)	**2.33(0.47,4.20)**	**4.20(2.40,6.00)**	**3.66(1.86,5.45)**	**4.18(2.38,5.99)**	**4.53(2.71,6.35)**	**3.46(1.63,5.30)**	**3.25(1.41,5.08)**
+O_3	**4.95(3.12,6.77)**	**4.08(2.32,5.84)**	**5.29(3.53,7.05)**	**4.60(2.82,6.39)**	**5.11(3.32,6.91)**	**5.43(3.63,7.24)**	**4.36(2.53,6.18)**	**3.95(2.13,5.78)**
**SO_2**	**5.60(3.27,7.93)**	**3.37(1.08,5.66)**	**4.20(1.91,6.48)**	**4.62(2.33,6.91)**	**5.98(3.70,8.27)**	**3.68(1.38,5.99)**	1.85(-0.45,4.16)	**2.38(0.08,4.67)**
+$PM_{2.5}$	**5.14(2.63,7.66)**	**2.89(0.57,5.22)**	**3.98(1.70,6.27)**	**4.47(2.19,6.76)**	**5.80(3.52,8.09)**	**3.45(1.14,5.76)**	1.58(-0.73,3.89)	2.13(-0.17,4.42)
+PM_{10}	**4.30(1.70,6.91)**	**2.36(0.01,4.71)**	**3.70(1.41,6.00)**	**4.26(1.97,6.54)**	**5.59(3.30,7.88)**	**3.22(0.91,5.52)**	1.31(-1.00,3.62)	1.82(-0.48,4.13)
+NO_2	**3.78(1.28,6.29)**	2.19(-0.14,4.52)	**3.64(1.36,5.92)**	**4.27(1.99,6.54)**	**5.75(3.48,8.03)**	**3.27(0.98,5.57)**	1.36(-0.93,3.66)	1.90(-0.38,4.19)
+CO	**3.32(0.63,6.00)**	2.00(-0.35,4.35)	**3.49(1.20,5.78)**	**4.22(1.95,6.50)**	**5.54(3.26,7.82)**	**3.10(0.80,5.40)**	1.30(-0.99,3.60)	1.99(-0.29,4.28)
+O_3	**5.69(3.37,8.01)**	**3.03(0.74,5.32)**	**3.88(1.59,6.16)**	**4.43(2.15,6.71)**	**5.79(3.51,8.07)**	**3.48(1.18,5.77)**	1.58(-0.72,3.88)	2.10(-0.19,4.39)
**NO_2**	**9.83(6.16,13.50)**	**9.92(6.41,13.44)**	**12.15(8.65,15.64)**	**12.56(9.03,16.09)**	**11.79(8.22,15.36)**	**12.27(8.70,15.84)**	**10.48(6.89,14.07)**	**8.57(4.96,12.18)**
+$PM_{2.5}$	**10.93(6.28,15.59)**	**9.76(5.82,13.71)**	**11.71(8.08,15.33)**	**12.08(8.49,15.67)**	**11.30(7.69,14.91)**	**11.79(8.18,15.41)**	**10.02(6.40,13.63)**	**8.17(4.55,11.79)**

+PM_{10}	**9.17(3.84,14.50)**	**8.48(4.25,12.70)**	**10.93(7.20,14.66)**	**11.42(7.76,15.07)**	**10.64(6.97,14.31)**	**11.16(7.49,14.84)**	**9.37(5.70,13.03)**	**7.50(3.84,11.16)**
+SO_2	**7.59(3.64,11.55)**	**8.49(4.90,12.08)**	**11.43(7.93,14.93)**	**12.04(8.51,15.56)**	**11.15(7.58,14.72)**	**11.63(8.06,15.19)**	**9.81(6.22,13.40)**	**7.98(4.38,11.58)**
+CO	**6.03(1.18,10.87)**	**7.00(2.97,11.04)**	**10.21(6.54,13.89)**	**10.93(7.31,14.55)**	**10.13(6.48,13.78)**	**10.62(6.96,14.28)**	**8.70(5.02,12.38)**	**6.91(3.24,10.57)**
+O3	**10.77(7.10,14.44)**	**9.90(6.40,13.40)**	**11.79(8.30,15.28)**	**12.22(8.70,15.75)**	**11.44(7.88,15.00)**	**11.85(8.29,15.42)**	**10.04(6.46,13.63)**	**8.12(4.51,11.73)**
**CO**	**17.81(11.11,24.51)**	**15.82(9.33,22.31)**	**18.70(12.23,25.18)**	**16.86(10.31,23.41)**	**18.64(12.05,25.24)**	**15.94(9.32,22.57)**	**8.62(1.96,15.29)**	**10.24(3.57,16.92)**
+$PM_{2.5}$	**17.41(9.81,25.00)**	**14.53(7.83,21.23)**	**17.78(11.23,24.33)**	**16.07(9.49,22.65)**	**17.90(11.28,24.53)**	**15.12(8.46,21.78)**	**7.93(1.25,14.60)**	**9.70(3.02,16.38)**
+PM_{10}	**14.60(6.65,22.55)**	**12.86(6.03,19.69)**	**16.69(10.09,23.28)**	**15.13(8.52,21.74)**	**17.03(10.38,23.68)**	**14.33(7.66,21.00)**	**7.11(0.42,13.80)**	**8.90(2.21,15.59)**
+SO_2	**12.97(5.22,20.71)**	**12.81(6.15,19.47)**	**16.76(10.24,23.28)**	**15.46(8.91,22.00)**	**17.00(10.4,23.61)**	**14.34(7.71,20.97)**	**6.87(0.20,13.54)**	**8.64(1.96,15.31)**
+NO_2	**10.58(1.74,19.43)**	**10.43(3.34,17.52)**	**14.95(8.23,21.68)**	**13.54(6.84,20.25)**	**16.00(9.29,22.70)**	**13.16(6.45,19.87)**	**5.68(-1.05,12.42)**	**7.66(0.94,14.38)**
+O_3	**17.50(10.83,24.18)**	**15.03(8.55,21.52)**	**17.83(11.36,24.31)**	**15.94(9.40,22.49)**	**17.66(11.07,24.26)**	**14.94(8.31,21.57)**	**7.65(0.99,14.31)**	**9.40(2.74,16.07)**
**O_3**	**-2.85(-4.25,-1.45)**	**-3.40(-4.73,-2.08)**	**-1.99(-3.22,-0.76)**	**-1.69(-2.87,-0.51)**	**-1.96(-3.12,-0.79)**	**-1.92(-3.06,-0.77)**	**-1.98(-3.10,-0.85)**	**-2.63(-3.74,-1.51)**
+$PM_{2.5}$	**-4.01(-5.50,-2.52)**	**-4.30(-5.68,-2.92)**	**-2.39(-3.63,-1.14)**	**-1.87(-3.05,-0.68)**	**-2.08(-3.25,-0.91)**	**-2.00(-3.15,-0.86)**	**-2.08(-3.21,-0.95)**	**-2.69(-3.81,-1.57)**
+PM_{10}	**-3.83(-5.27,-2.40)**	**-4.20(-5.55,-2.85)**	**-2.28(-3.51,-1.05)**	**-1.74(-2.91,-0.56)**	**-1.93(-3.09,-0.77)**	**-1.86(-3.00,-0.72)**	**-1.93(-3.06,-0.81)**	**-2.54(-3.65,-1.42)**
+SO_2	**-2.85(-4.24,-1.47)**	**-3.25(-4.57,-1.92)**	**-1.86(-3.08,-0.63)**	**-1.56(-2.74,-0.38)**	**-1.75(-2.92,-0.59)**	**-1.72(-2.86,-0.58)**	**-1.79(-2.92,-0.67)**	**-2.41(-3.53,-1.30)**
+NO_2	**-3.24(-4.63,-1.85)**	**-3.82(-5.14,-2.50)**	**-1.96(-3.18,-0.74)**	**-1.46(-2.64,-0.29)**	**-1.63(-2.79,-0.46)**	**-1.60(-2.75,-0.46)**	**-1.66(-2.79,-0.53)**	**-2.28(-3.41,-1.16)**
+CO	**-2.70(-4.09,-1.32)**	**-3.21(-4.53,-1.89)**	**-1.77(-3.00,-0.55)**	**-1.40(-2.58,-0.22)**	**-1.63(-2.79,-0.46)**	**-1.61(-2.75,-0.46)**	**-1.70(-2.83,-0.58)**	**-2.40(-3.51,-1.28)**

Notes: Bold fonts indicates the effect was statistically significant ($P<0.05$).

Table S16 Absolute increase (95% CI） in hospital cost for RD-DM associated with one unit increase in $PM_{2.5}$, PM_{10}, SO_2, NO_2, CO and O_3 along different lag days using single-pollutant and two-pollutant models.

	Lag0	Lag1	Lag2	Lag3	Lag4	Lag5	Lag6	Lag7
**$PM_{2.5}$**	3354.18<br>(-77.40,6785.76)	3134.39<br>(-301.00,6569.78)	**5087.99**<br>**(1630.72,8545.26)**	**4484.84**<br>**(989.54,7980.15)**	**6844.49**<br>**(3326.77,10362.21)**	**8339.03**<br>**(4810.89,11867.17)**	**6873.65**<br>**(3323.38,10423.92)**	**5644.43**<br>**(2069.06,9219.80)**
+SO_2	1058.62<br>(-2625.28,4742.52)	2224.65<br>(-1237.13,5686.43)	**4710.33**<br>**(1259.50,8161.15)**	**4246.93**<br>**(763.51,7730.34)**	**6491.25**<br>**(2980.54,10001.95)**	**7899.92**<br>**(4375.71,11424.13)**	**6309.50**<br>**(2756.34,9862.66)**	**5013.65**<br>**(1432.70,8594.60)**
+NO_2	-2052.69<br>(-6384.36,2278.98)	123.82<br>(-3616.86,3864.49)	3447.94<br>(-105.10,7000.98)	3123.83<br>(-427.28,6674.94)	**5603.30**<br>**(2037.08,9169.52)**	**7216.02**<br>**(3642.74,10789.3)**	**5866.77**<br>**(2289.16,9444.38)**	**4769.13**<br>**(1180.81,8357.44)**
+CO	-679.51<br>(-4542.92,3183.90)	565.72<br>(-3040.10,4171.53)	3652.00<br>(149.00,7155.00)	3457.13<br>(-48.71,6962.97)	**5789.98**<br>**(2257.52,9322.44)**	**7214.26**<br>**(3662.73,10765.79)**	**5664.31**<br>**(2086.18,9242.43)**	**4452.61**<br>**(854.92,8050.30)**
+O_3	**6058.52**<br>**(2403.22,9713.82)**	**4213.72**<br>**(730.60,7696.83)**	**5891.40**<br>**(2412.91,9369.88)**	**5342.95**<br>**(1822.21,8863.69)**	**7554.99**<br>**(4014.1,11095.89)**	**8925.48**<br>**(5388.08,12462.88)**	**7398.74**<br>**(3846.47,10951.01)**	**6008.16**<br>**(2437.11,9579.20)**
**PM_{10}**	**3476.58**<br>**(1320.2,5632.97)**	**3373.05**<br>**(1225.94,5520.16)**	**4876.06**<br>**(2717.68,7034.44)**	**3891.08**<br>**(1704.42,6077.73)**	**5376.26**<br>**(3180.42,7572.10)**	**5519.42**<br>**(3313.67,7725.16)**	**4621.06**<br>**(2398.55,6843.58)**	**3981.96**<br>**(1748.55,6215.37)**
+SO_2	1982.61<br>(-424.34,4389.56)	**2674.38**<br>**(492.60,4856.17)**	**4500.69**<br>**(2337.37,6664.01)**	**3556.11**<br>**(1368.53,5743.69)**	**4988.92**<br>**(2788.24,7189.61)**	**5118.29**<br>**(2906.01,7330.57)**	**4158.00**<br>**(1924.41,6391.58)**	**3543.15**<br>**(1301.49,5784.82)**
+NO_2	-23.80<br>(-3150.33,3102.73)	1453.39<br>(-973.55,3880.32)	**3782.28**<br>**(1524.23,6040.33)**	**2933.00**<br>**(691.21,5174.78)**	**4541.18**<br>**(2299.62,6782.75)**	**4729.75**<br>**(2480.2,6979.31)**	**3902.72**<br>**(1651.90,6153.53)**	**3338.42**<br>**(1087.42,5589.41)**
+CO	878.18<br>(-1671.38,3427.73)	1801.92<br>(-471.19,4075.02)	**3955.42**<br>**(1758.45,6152.38)**	**3219.33**<br>**(1023.04,5415.61)**	**4731.10**<br>**(2525.72,6936.49)**	**4824.60**<br>**(2604.01,7045.18)**	**3918.86**<br>**(1682.33,6155.39)**	**3368.06**<br>**(1128.34,5607.78)**
+O_3	**4587.53**<br>**(2352.59,6822.47)**	**3679.22**<br>**(1523.31,5835.13)**	**5096.30**<br>**(2938.44,7254.17)**	**4177.47**<br>**(1989.31,6365.63)**	**5641.59**<br>**(3441.76,7841.41)**	**5716.59**<br>**(3511.38,7921.80)**	**4845.04**<br>**(2624.98,7065.10)**	**4106.07**<br>**(1877.57,6334.57)**
**SO_2**	**5565.81**<br>**(2723.86,8407.77)**	**3276.85**<br>**(487.19,6066.51)**	**5379.51**<br>**(2598.21,8160.81)**	**5309.23**<br>**(2521.68,8096.78)**	**6450.01**<br>**(3662.28,9237.75)**	**4123.40**<br>**(1319.74,6927.05)**	1416.87<br>(-1382.87,4216.62)	**2867.45**<br>**(78.90,5656.00)**

+$PM_{2.5}$	**5230.32 (2167.17,8293.48)**	**2878.11 (44.76,5711.45)**	**5218.75 (2432.69,8004.81)**	**5203.06 (2415.75,7990.37)**	**6292.00 (3500.68,9083.31)**	**3943.00 (1133.70,6752.30)**	1188.09 (-1619.39,3995.56)	2663.83 (-131.40,5459.06)
+PM_{10}	**4370.63 (1197.18,7544.08)**	2355.89 (-507.57,5219.36)	**4950.19 (2158.48,7741.91)**	**4995.55 (2206.91,7784.19)**	**6078.49 (3284.95,8872.03)**	**3732.32 (921.56,6543.07)**	941.98 (-1868.73,3752.69)	2391.16 (-410.64,5192.96)
+NO_2	**3701.99 (641.64,6762.33)**	2128.51 (-710.95,4967.96)	**4853.63 (2071.37,7635.89)**	**5019.47 (2238.01,7800.92)**	**6221.55 (3443.85,8999.25)**	**3757.35 (959.73,6554.96)**	965.16 (-1832.36,3762.67)	2420.43 (-366.03,5206.90)
+CO	2710.27 (-559.29,5979.83)	1735.93 (-1119.08,4590.95)	**4610.20 (1824.49,7395.90)**	**4877.15 (2101.81,7652.48)**	**5937.40 (3157.67,8717.13)**	**3494.25 (697.24,6291.26)**	807.01 (-1982.49,3596.51)	2443.16 (-331.64,5217.96)
+O_3	**5452.74 (2609.67,8295.81)**	**2825.84 (29.75,5621.94)**	**4977.33 (2190.25,7764.40)**	**5019.63 (2231.33,7807.92)**	**6122.65 (3328.05,8917.25)**	**3830.49 (1027.34,6633.64)**	1091.50 (-1707.57,3890.57)	2552.51 (-235.45,5340.47)
**NO_2**	**9962.69 (5486.53,14438.86)**	**10412.57 (6121.63,14703.52)**	**12482.80 (8209.44,16756.16)**	**12416.43 (8094.06,16738.81)**	**12284.51 (7920.52,16648.5)**	**14646.23 (10298.76,18993.70)**	**12542.16 (8171.70,16912.61)**	**10143.88 (5749.33,14538.42)**
+$PM_{2.5}$	**11632.80 (5951.61,17313.98)**	**10630.18 (5815.50,15444.85)**	**12192.87 (7756.68,16629.06)**	**12010.87 (7616.73,16405.02)**	**11871.86 (7454.25,16289.47)**	**14303.35 (9901.61,18705.09)**	**12170.43 (7764.84,16576.03)**	**9800.36 (5387.58,14213.13)**
+PM_{10}	**10001.89 (3498.58,16505.2)**	**9442.56 (4285.70,14599.41)**	**11438.35 (6876.54,16000.15)**	**11349.35 (6874.5,15824.21)**	**11219.53 (6730.38,15708.68)**	**13770.33 (9297.21,18243.45)**	**11589.94 (7127.54,16052.35)**	**9176.44 (4717.61,13635.27)**
+SO_2	**7798.46 (2971.47,12625.44)**	**9030.31 (4639.31,13421.30)**	**11785.66 (7501.66,16069.67)**	**11887.39 (7566.36,16208.42)**	**11627.86 (7257.61,15998.11)**	**14041.67 (9689.48,18393.85)**	**11916.29 (7541.15,16291.44)**	**9592.66 (5198.97,13986.35)**
+CO	4811.78 (-1083.61,10707.17)	**6854.44 (1934.11,11774.76)**	**10152.84 (5663.33,14642.34)**	**10447.39 (6013.87,14880.91)**	**10331.02 (5862.05,14800.00)**	**12823.05 (8365.93,17280.17)**	**10568.44 (6086.34,15050.54)**	**8271.66 (3804.41,12738.91)**
+O3	**10628.14 (6114.59,15141.69)**	**10174.2 (5871.1,14477.3)**	**11998.77 (7709.27,16288.27)**	**11987.79 (7651.62,16323.96)**	**11883.40 (7509.36,16257.44)**	**14247.03 (9894.13,18599.93)**	**11972.30 (7583.33,16361.28)**	**9487.97 (5075.38,13900.57)**
**CO**	**20101.01 (11930.66,28271.35)**	**15017.11 (7089.94,22944.27)**	**19502.84 (11594.37,27411.3)**	**18419.57 (10425.74,26413.39)**	**18468.18 (10403.15,26533.21)**	**19294.78 (11232.67,27356.88)**	**13285.59 (5178.76,21392.42)**	**13903.67 (5779.12,22028.23)**

+$PM_{2.5}$	**20870.82 (11607.39,30134.26)**	**13882.87 (5698.65,22067.10)**	**18692.87 (10691.41,26694.34)**	**17738.30 (9696.94,25779.66)**	**17808.86 (9701.92,25915.81)**	**18617.17 (10504.07,26730.26)**	**12730.73 (4603.21,20858.25)**	**13468.16 (5335.01,21601.31)**
+PM_{10}	**18301.13 (8604.24,27998.03)**	**12167.33 (3820.83,20513.82)**	**17612.36 (9549.33,25675.39)**	**16816.81 (8736.16,24897.47)**	**16924.74 (8783.67,25065.81)**	**17850.98 (9717.11,25984.86)**	**11958.66 (3810.83,20106.48)**	**12704.25 (4551.35,20857.15)**
+SO_2	**16115.01 (6684.91,25545.12)**	**11951.46 (3810.88,20092.04)**	**17499.10 (9523.75,25474.45)**	**16958.65 (8948.87,24968.43)**	**16805.97 (8709.09,24902.85)**	**17705.18 (9619.60,25790.76)**	**11609.54 (3482.59,19736.49)**	**12353.41 (4216.42,20490.40)**
+NO_2	**14300.36 (3523.56,25077.16)**	**9307.95 (649.46,17966.44)**	**15665.95 (7439.17,23892.72)**	**15073.67 (6879.51,23267.83)**	**15569.65 (7378.41,23760.9)**	**16556.33 (8378.48,24734.18)**	**10448.71 (2245.12,18652.3)**	**11369.72 (3178.42,19561.03)**
+O_3	**19326.07 (11144.9,27507.24)**	**13726.15 (5759.15,21693.14)**	**18326.82 (10361.76,26291.87)**	**17215.59 (9155.68,25275.51)**	**17172.84 (9035.31,25310.36)**	**17989.55 (9864.87,26114.23)**	**11810.05 (3643.83,19976.28)**	**12575.34 (4403.55,20747.13)**
**O_3**	**-2880.98 (-4602.23,-1159.72)**	**-3912.03 (-5533.27,-2290.80)**	**-2542.07 (-4039.62,-1044.52)**	**-2226.42 (-3668.39,-784.44)**	**-2399.29 (-3821.90,-976.68)**	**-2520.44 (-3915.16,-1125.71)**	**-2441.72 (-3817.20,-1066.24)**	**-3406.86 (-4766.1,-2047.62)**
+$PM_{2.5}$	**-3945.59 (-5779.79,-2111.38)**	**-4749.75 (-6437.27,-3062.22)**	**-2940.52 (-4462.16,-1418.87)**	**-2415.81 (-3861.43,-970.19)**	**-2529.34 (-3952.58,-1106.10)**	**-2609.03 (-4003.17,-1214.89)**	**-2549.17 (-3925.26,-1173.08)**	**-3465.68 (-4824.53,-2106.83)**
+PM_{10}	**-3763.78 (-5533.04,-1994.52)**	**-4626.36 (-6276.16,-2976.55)**	**-2846.11 (-4346.74,-1345.49)**	**-2293.10 (-3729.75,-856.45)**	**-2392.73 (-3810.56,-974.89)**	**-2476.41 (-3867.43,-1085.38)**	**-2408.27 (-3780.00,-1036.53)**	**-3322.15 (-4679.61,-1964.68)**
+SO_2	**-2867.63 (-4579.47,-1155.79)**	**-3727.24 (-5345.30,-2109.18)**	**-2414.74 (-3909.02,-920.46)**	**-2111.47 (-3548.44,-674.51)**	**-2209.20 (-3630.10,-788.30)**	**-2335.49 (-3728.64,-942.34)**	**-2267.04 (-3640.65,-893.43)**	**-3202.05 (-4562.18,-1841.91)**
+NO_2	**-3172.14 (-4883.65,-1460.63)**	**-4246.22 (-5862.73,-2629.71)**	**-2517.00 (-4007.03,-1026.96)**	**-2012.60 (-3450.63,-574.58)**	**-2077.77 (-3502.68,-652.86)**	**-2215.52 (-3612.77,-818.28)**	**-2129.77 (-3508.29,-751.25)**	**-3070.70 (-4436.76,-1704.64)**
+CO	**-2687.18 (-4395.95,-978.41)**	**-3653.30 (-5268.17,-2038.44)**	**-2302.29 (-3795.19,-809.39)**	**-1913.36 (-3352.67,-474.05)**	**-2042.40 (-3465.74,-619.05)**	**-2174.91 (-3569.90,-779.93)**	**-2135.48 (-3509.37,-761.59)**	**-3143.75 (-4500.74,-1786.76)**

Notes: Bold fonts indicates the effect was statistically significant (P<0.05).

Table S17 The admissions attributable risk for different population with different air quality guideline by gender group.

Type	Air Pollution	Guideline	Male		Female	
			Avoidable value	Attributable fraction (%)	Avoidable value	Attributable fraction (%)
**WHO 2021**	$PM_{2.5}$	15	1237(806,1637)	11.47(7.47,15.18)	372(5,705)	5.69(0.08,10.79)
	PM_{10}	45	826(604,1035)	7.66(5.60,9.59)	242(55,415)	3.70(0.84,6.34)
	SO_2	40	297(222,367)	2.75(2.06,3.40)	124(63,180)	1.89(0.97,2.75)
	NO_2	25	1303(980,1604)	12.08(9.09,14.87)	508(234,758)	7.77(3.59,11.60)
	CO	4	8(6,9)	0.07(0.05,0.09)	2(1,3)	0.03(0.02,0.04)
**Threshold concentration = 0**	$PM_{2.5}$	0	2394(1585,3122)	22.20(14.69,28.95)	742(10,1380)	11.36(0.16,21.12)
	PM_{10}	0	3258(2445,3982)	30.21(22.67,36.93)	1024(242,1697)	15.67(3.71,25.97)
	SO_2	0	3170(2422,3841)	29.39(22.45,35.61)	1386(726,1960)	21.21(11.11,29.99)
	NO_2	0	3919(3033,4694)	36.33(28.13,43.53)	1614(774,2321)	24.70(11.85,35.52)
	CO	0	3289(2482,4008)	30.50(23.02,37.17)	1446(718,2073)	22.13(10.99,31.73)

Notes: The association between O_3 and admissions was negative, so we did not calculate the attributable risk.

Table S18 The admissions attributable risk for different population with different air quality guideline by age group.

Type	Air Pollution	Guideline	Younger Adult		The elderly	
			Avoidable value	Attributable fraction (%)	Avoidable value	Attributable fraction (%)
**WHO 2021**	$PM_{2.5}$	15	227(-156,574)	3.52(-2.41,8.89)	1390(963,1788)	12.79(8.86,16.45)
	PM_{10}	45	145(-46,321)	2.25(-0.72,4.98)	919(697,1129)	8.46(6.41,10.39)
	SO_2	40	102(43,155)	1.58(0.67,2.41)	316(239,388)	2.91(2.20,3.57)
	NO_2	25	193(-108,467)	2.99(-1.68,7.24)	1565(1256,1855)	14.41(11.56,17.07)
	CO	4	3(1,4)	0.04(0.01,0.06)	7(5,8)	0.06(0.05,0.07)
**Threshold concentration = 0**	$PM_{2.5}$	0	463(-324,1145)	7.17(-5.03,17.75)	2660(1871,3369)	24.48(17.22,31.01)
	PM_{10}	0	649(-215,1389)	10.06(-3.33,21.52)	3523(2739,4223)	32.42(25.21,38.86)
	SO_2	0	1216(531,1810)	18.84(8.23,28.05)	3264(2520,3931)	30.03(23.19,36.18)
	NO_2	0	662(-388,1538)	10.26(-6.01,23.83)	4533(3737,5232)	41.71(34.39,48.15)
	CO	0	1207(423,1878)	18.71(6.56,29.10)	3502(2717,4204)	32.23(25.00,38.68)

Notes: The association between O_3 and admissions was negative, so we did not calculate the attributable risk; Younger Adult: Age less than or equal to 65 years old; The elderly: Age over 65 years old

Table S19 The LOS attributable risk for different population with different air quality guideline by gender group.

Type	Air Pollution	Guideline	Male		Female	
			Avoidable value	Attributable fraction (%)	Avoidable value	Attributable fraction (%)
**WHO 2021**	$PM_{2.5}$	15	24207(15596,32818)	16.82(10.83,22.80)	6283(1354,11213)	9.09(1.96,16.22)
	PM_{10}	45	14388(10176,18600)	10.00(7.07,12.92)	3813(1392,6234)	5.52(2.01,9.02)
	SO_2	40	3748(2302,5194)	2.60(1.60,3.61)	1486(663,2310)	2.15(0.96,3.34)
	NO_2	25	24543(18016,31070)	17.05(12.52,21.58)	8822(5046,12599)	12.76(7.3,18.22)
	CO	4	50(34,66)	0.03(0.02,0.05)	19(10,29)	0.03(0.01,0.04)
**Threshold concentration = 0**	$PM_{2.5}$	0	50580(32588,68572)	35.14(22.64,47.64)	13129(2828,23429)	18.99(4.09,33.89)
	PM_{10}	0	68453(48415,88492)	47.55(33.63,61.47)	18141(6623,29658)	26.24(9.58,42.90)
	SO_2	0	48970(30074,67866)	34.02(20.89,47.15)	19419(8657,30180)	28.09(12.52,43.66)
	NO_2	0	89518(65710,113325)	62.19(45.65,78.72)	32178(18404,45952)	46.55(26.62,66.47)
	CO	0	63286(42914,83658)	43.96(29.81,58.12)	24716(12948,36485)	35.75(18.73,52.78)

Notes: The association between O_3 and LOS was negative, so we did not calculate the attributable risk; LOS: length of hospital stay.

Table S20 The LOS attributable risk for different population with different air quality guideline by age group.

Type	Air Pollution	Guideline	Younger Adult		The elderly	
			Avoidable value	Attributable fraction (%)	Avoidable value	Attributable fraction (%)
**WHO 2021**	$PM_{2.5}$	15	5205(998,9412)	8.55(1.64,15.47)	25557(16204,34910)	16.79(10.64,22.93)
	PM_{10}	45	2406(342,4470)	3.95(0.56,7.35)	16039(11466,20612)	10.54(7.53,13.54)
	SO_2	40	933(250,1616)	1.53(0.41,2.66)	4301(2732,5870)	2.83(1.79,3.86)
	NO_2	25	3215(8,6423)	5.28(0.01,10.56)	30563(23509,37616)	20.08(15.44,24.71)
	CO	4	14(6,22)	0.02(0.01,0.04)	57(40,75)	0.04(0.03,0.05)
**Threshold concentration = 0**	$PM_{2.5}$	0	10876(2086,19666)	17.87(3.43,32.32)	53401(33858,72943)	35.08(22.24,47.92)
	PM_{10}	0	11447(1628,21265)	18.81(2.68,34.95)	76308(54549,98066)	50.13(35.83,64.42)
	SO_2	0	12191(3267,21116)	20.04(5.37,34.70)	56196(35696,76696)	36.91(23.45,50.38)
	NO_2	0	11727(28,23427)	19.27(0.05,38.50)	111473(85747,137199)	73.23(56.33,90.12)
	CO	0	17717(7630,27803)	29.12(12.54,45.69)	72803(50714,94893)	47.82(33.31,62.33)

Notes: The association between O_3 and LOS was negative, so we did not calculate the attributable risk; LOS: length of hospital stay; Younger Adult: Age less than or equal to 65 years old; The elderly: Age over 65 years old

Table S21 The hospital cost attributable risk for different population with different air quality guideline by gender group.

Type	Air Pollution	Guideline	Male		Female	
			Avoidable value	Attributable fraction (%)	Avoidable value	Attributable fraction (%)
**WHO 2021**	$PM_{2.5}$	15	26457977 (15361564,37554390)	18.22(10.58,25.86)	3666101 (-2000837,9333040)	5.89(-3.21,14.98)
	PM_{10}	45	16351525 (10933310,21769740)	11.26(7.53,14.99)	2246207 (-537958,5030372)	3.61(-0.86,8.08)
	SO_2	40	4665884 (2807761,6524006)	3.21(1.93,4.49)	1038651 (95037,1982265)	1.67(0.15,3.18)
	NO_2	25	29444914 (21036953,37852875)	20.28(14.49,26.07)	6282831 (1963670,10601992)	10.09(3.15,17.02)
	CO	4	67389(46926,87851)	0.05(0.03,0.06)	11513(920,22106)	0.02(0.00,0.04)
**Threshold concentration = 0**	$PM_{2.5}$	0	55282942 (32097408,78468477)	38.07(22.10,54.03)	7660180 (-4180673,19501034)	12.30(-6.71,31.31)
	PM_{10}	0	77794714 (52016782,103572646)	53.57(35.82,71.32)	10686651 (-2559412,23932715)	17.16(-4.11,38.42)
	SO_2	0	60962617 (36685112,85240122)	41.98(25.26,58.70)	13570608 (1241710,25899506)	21.79(1.99,41.58)
	NO_2	0	107396301 (76729413,138063190)	73.95(52.84,95.07)	22915769 (7162219,38669319)	36.79(11.50,62.08)
	CO	0	85677748 (59661416,111694081)	59.00(41.08,76.91)	14637373 (1169057,28105690)	23.50(1.88,45.12)

Notes: The association between O_3 and hospital cost was negative, so we did not calculate the attributable risk...

Table S22 The hospital cost attributable risk for different population with different air quality guideline by age group.

Type	Air Pollution	Guideline	Younger Adult		The elderly	
			**Avoidable value**	**Attributable fraction (%)**	**Avoidable value**	**Attributable fraction (%)**
**WHO 2021**	$PM_{2.5}$	15	5392636 (248956,10536316)	9.45(0.44,18.47)	24789716 (13021302,36558131)	16.48(8.65,24.30)
	PM_{10}	45	2249833 (-273324,4772990)	3.94(-0.48,8.37)	16314845 (10553785,22075906)	10.84(7.01,14.67)
	SO_2	40	1022044(191094,1852994)	1.79(0.34,3.25)	4714277 (2746733,6681822)	3.13(1.83,4.44)
	NO_2	25	2549798 (-1338624,6438220)	4.47(-2.35,11.29)	33415660 (24526258,42305061)	22.21(16.30,28.12)
	CO	4	13896(4302,23490)	0.02(0.01,0.04)	64144(42265,86022)	0.04(0.03,0.06)
**Threshold concentration = 0**	$PM_{2.5}$	0	11267709 (520185,22015234)	19.75(0.91,38.60)	51797175 (27207518,76386831)	34.42(18.08,50.77)
	PM_{10}	0	10703901 (-1300378,22708181)	18.77(-2.28,39.81)	77620205 (50211136,105029274)	51.59(33.37,69.80)
	SO_2	0	13353628 (2496759,24210498)	23.41(4.38,42.44)	61594910 (35887741,87302078)	40.94(23.85,58.02)
	NO_2	0	9300040 (-4882450,23482529)	16.30(-8.56,41.17)	121879054 (89456176,154301932)	81.00(59.45,102.55)
	CO	0	17667197 (5469081,29865313)	30.97(9.59,52.36)	81552625 (53736316,109368935)	54.20(35.71,72.69)

Notes: The association between O_3 and hospital cost was negative, so we did not calculate the attributable risk; Younger Adult: Age less than or equal to 65 years old; The elderly: Age over 65 years old.

Table S23 Results of E value analysis for association between air pollutants with RD-DM admissions in different lag days.

Air pollutant	lag days	RR	E value	Stable*
**$PM_{2.5}$**	0	1.013	1.000	No
	1	1.019	1.000	No
	2	1.034	1.136	Yes
	3	1.032	1.126	Yes
	4	1.042	1.171	Yes
	5	1.048	1.199	Yes
	6	1.039	1.156	Yes
	7	1.035	1.138	Yes
	01	1.019	1.000	No
	02	1.030	1.097	Yes
	03	1.038	1.133	Yes
	04	1.048	1.174	Yes
	05	1.059	1.214	Yes
	06	1.066	1.236	Yes
	07	1.072	1.253	Yes
**PM_{10}**	0	1.017	1.082	Yes
	1	1.021	1.103	Yes
	2	1.031	1.156	Yes
	3	1.028	1.142	Yes
	4	1.031	1.158	Yes
	5	1.030	1.154	Yes
	6	1.025	1.128	Yes
	7	1.024	1.122	Yes
	01	1.023	1.110	Yes
	02	1.032	1.152	Yes
	03	1.038	1.175	Yes
	04	1.044	1.198	Yes
	05	1.049	1.216	Yes
	06	1.053	1.227	Yes
	07	1.056	1.236	Yes
SO_2	0	1.040	1.181	Yes
	1	1.025	1.106	Yes
	2	1.028	1.128	Yes
	3	1.038	1.177	Yes
	4	1.049	1.220	Yes
	5	1.038	1.173	Yes
	6	1.022	1.093	Yes
	7	1.022	1.087	Yes
	01	1.046	1.196	Yes
	02	1.056	1.225	Yes
	03	1.071	1.270	Yes
	04	1.089	1.325	Yes
	05	1.099	1.352	Yes
	06	1.100	1.352	Yes
	07	1.101	1.353	Yes
**NO_2**	0	1.048	1.179	Yes

	1	1.055	1.207	Yes
	2	1.070	1.263	Yes
	3	1.073	1.272	Yes
	4	1.075	1.278	Yes
	5	1.076	1.282	Yes
	6	1.064	1.241	Yes
	7	1.058	1.218	Yes
	01	1.060	1.220	Yes
	02	1.076	1.272	Yes
	03	1.090	1.312	Yes
	04	1.102	1.344	Yes
	05	1.112	1.372	Yes
	06	1.119	1.388	Yes
	07	1.123	1.398	Yes
**CO**	0	1.102	1.302	Yes
	1	1.106	1.318	Yes
	2	1.122	1.366	Yes
	3	1.101	1.299	Yes
	4	1.125	1.367	Yes
	5	1.109	1.319	Yes
	6	1.072	1.197	Yes
	7	1.068	1.179	Yes
	01	1.136	1.385	Yes
	02	1.172	1.470	Yes
	03	1.194	1.515	Yes
	04	1.224	1.579	Yes
	05	1.246	1.622	Yes
	06	1.252	1.631	Yes
	07	1.258	1.638	Yes
**O_3**	0	0.981	1.105	Yes
	1	0.978	1.129	Yes
	2	0.983	1.099	Yes
	3	0.988	1.067	Yes
	4	0.985	1.094	Yes
	5	0.985	1.095	Yes
	6	0.985	1.097	Yes
	7	0.980	1.125	Yes
	01	0.974	1.142	Yes
	02	0.973	1.147	Yes
	03	0.974	1.141	Yes
	04	0.973	1.145	Yes
	05	0.972	1.149	Yes
	06	0.972	1.151	Yes
	07	0.970	1.160	Yes

Notes: *Stable was considered as the RR value less than the E value.

Table S24 Time series data example for RD-DM patients

Outcome	DATE	Total	<65	>=65	Male	Female
Admissions	2016/1/1	4	0	4	4	0
	2016/1/2	7	2	5	4	3
	2016/1/3	12	2	10	6	6
	2016/1/4	16	6	10	10	6
	2016/1/5	15	5	10	10	5
	……	……	……	……	……	……
	……	……	……	……	……	……
LOS# (Days)	2016/1/1	55	0	55	55	0
	2016/1/2	65	11	54	41	24
	2016/1/3	160	21	139	101	59
	2016/1/4	220	29	191	88	132
	2016/1/5	244	64	180	204	40
	……	……	……	……	……	……
	……	……	……	……	……	……
Hospital cost (Yuan)	2016/1/1	34001.27	0	34001.27	34001.27	0
	2016/1/2	47833.71	8366.85	39466.86	29136.38	18697.33
	2016/1/3	134257.61	13933.05	120324.56	92952.93	41304.68
	2016/1/4	148612.72	22623.36	125989.36	83520.79	65091.93
	2016/1/5	351563.73	88119.64	263444.09	324111.46	27452.27
	……	……	……	……	……	……
	……	……	……	……	……	……

Notes: # LOS: length of stay
